# The Role of the Tyrosine-Based Sorting Signals of the ORF3a Protein of SARS-CoV-2 in Intracellular Trafficking and Pathogenesis

**DOI:** 10.3390/v17040522

**Published:** 2025-04-03

**Authors:** Edward B. Stephens, Dusan Kunec, Wyatt Henke, Ricardo Martin Vidal, Brandon Greishaber, Rabina Saud, Maria Kalamvoki, Gagandeep Singh, Sujan Kafle, Jessie D. Trujillo, Franco Matias Ferreyra, Igor Morozov, Juergen A. Richt

**Affiliations:** 1 Department of Microbiology, Molecular Genetics, and Immunology 2000 Cates Hall, University of Kansas Medical Center, 3901 Rainbow Blvd, Kansas City, KS 66160, USA; 2 Institut für Virologie, Freie Universität Berlin, Berlin, Germany; kunec.dusan@fu-berlin.de (D.K.);; 3 Department of Diagnostic Medicine/Pathobiology, College of Veterinary Medicine, Kansas State University, Manhattan, KS 66506, USA; gdsdhol@vet.k-state.edu (G.S.); jdtrujillo@vet.k-state.edu (J.D.T.);; 4 Veterinary Diagnostic Laboratory, College of Veterinary Medicine, Kansas State University, Manhattan, KS 66506, USA

**Keywords:** SARS-CoV-2, ORF3a, tyrosine-based sorting motifs, intracellular transport, viroporin, viral pathogenesis

## Abstract

The open reading frame 3a (ORF3a) is a protein important to the pathogenicity of SARS-CoV-2. The cytoplasmic domain of ORF3a has three canonical tyrosine-based sorting signals (160YNSV163, 211YYQL213, and 233YNKI236), and a previous study has indicated that mutation of the 160YNSV163 motif abrogated plasma membrane expression and inhibited ORF3a-induced apoptosis. Here, we have systematically removed all three tyrosine-based motifs and assessed the importance of each motif or combination of motifs in trafficking to the cell surface. Our results indicate that the 160YNSV163 motif alone was insufficient for ORF3a cell-surface trafficking, while the 211YYQL213 motif was the most important. Additionally, an ORF3a with all three YxxΦ motifs disrupted (ORF3a-[ΔYxxΦ]) was not transported to the cell surface, and LysoIP studies indicate that ORF3a but not ORF3a-[ΔYxxΦ] was present in late endosome/lysosome fractions. A growth-curve analysis of different SARS-CoV-2 viruses expressing the different mutant ORF3a proteins revealed no significant differences in virus replication. Finally, the inoculation of K18hACE-2 mice indicated that the SARS-CoV-2 lacking the three YxxΦ motifs was less pathogenic than the unmodified SARS-CoV-2. These results indicate that the tyrosine motifs of ORF3a contribute to cell-surface expression and SARS-CoV-2 pathogenesis

## 1. Introduction

First identified in SARS-CoV, the ORF3a protein is the largest accessory protein of SARS-CoV and SARS-CoV-2 at 276 and 275 amino acids, respectively. These proteins have N-terminal domains of approximately 40 amino acids, three transmembrane domains, and a longer cytoplasmic domain of approximately 150 amino acids [1]. The ORF3a proteins from SARS-CoV and SARS-CoV-2 form multimeric structures in membranes that have been reported to function as ion channels (i.e., viroporins), previously shown to be permissive to divalent cations [1,2,3,4,5]. The SARS-CoV-2 ORF3a behaved as a cation channel with a sizeable single-channel conductance (375 pA) and had a modest selectivity for Ca^++^ and K^+^ over Na^+^. Both the SARS-CoV and SARS-CoV-2 ORF3a ion channels were blocked by Ba^++^, suggesting that these may be potassium-sensitive ion channels [4,6]. However, the data from a more recent study that has examined the ion channel activity of ORF3a protein in different systems, including mammalian cell lines, suggest that the ORF3a may not be a viroporin [5,7,8].

Previous studies have shown that ORF3a can be detected within the ER, ERGIC, Golgi complex, trans-Golgi network (TGN), lysosomes, and the cell-plasma membrane. Previous studies have established that linear sorting signals mediate the trafficking of cellular membrane proteins from the Golgi complex to their final membrane compartment. Most sorting occurs within the TGN [9]. These short linear sequences include the (a) dileucine motifs ([D/E]xxxL[L/I] or [D/E]xxL[L/I]; (b) tyrosine-based motifs (YxxΦ; with x being any amino acid and Φ being an amino acid with a large hydrophobic -R group) and are both recognized by the adaptor protein complexes AP-1, AP-2, and AP-3; and (c) Asn-Pro-X-Tyr (NPXY) motifs, which are recognized by the accessory clathrin adaptor proteins [9,10,11]. The YxxΦ sorting signals have dual specificity, directing the trafficking of membrane proteins within the endosomal and secretory pathways and in rapid endocytosis from the cell surface and sorting to lysosomes and lysosome-related organelles [12,13,14,15,16,17,18,19,20,21,22,23,24,25].

Like cellular membrane proteins, many viral membrane proteins including those from the orthomyxoviruses, herpesviruses, retroviruses, and other coronaviruses rely on the use of canonical tyrosine-based signals for intracellular transport, and mutation of these sites can affect the trafficking of these proteins and their biological activity within the cell [24,25,26,27,28,29,30]. The membrane (M) protein and accessory protein ORF3a, which have YxxΦ motifs, are no exception. Previous studies on the ORF3a proteins of SARS-CoV and SARS-CoV-2 have shown that the mutation of the tyrosine residue in motif ^160^YNSV^163^ resulted in an ORF3a protein that was not present at the cell surface [31,32]. Further, these investigators showed that the lack of cell-surface expression correlated with reduced apoptosis [32]. However, the cytoplasmic domains from ORF3a proteins from the SARS-CoV, SARS-CoV-2, and various related bat coronaviruses within the genus Sarbecovirus of the β-coronaviruses have two to three well-conserved YxxΦ motifs in the cytoplasmic domain. Our detailed analysis of the three potential YxxΦ-based sorting motifs of the SARS-CoV-2 ORF3a revealed that the ^211^YYQL^214^ motif was crucial in targeting ORF3a to the cell-plasma membrane. Further, we show that removing the tyrosine-based sorting motifs decreases the pathogenicity of SARS-CoV-2.

## 2. Materials and Methods

### 2.1. Cells, Viruses, and Plasmids

HEK293 and COS-7 cells were used to transfect vectors expressing ORF3a proteins. Both cell lines were maintained in Dulbecco’s minimal essential medium (DMEM) with 10% fetal bovine serum, 10 mM Hepes buffer, pH 7.3, 100 U/mL penicillin, 100 μg/mL streptomycin, and 5 μg gentamicin. Plasmids (all pcDNA3.1(+) based) expressing the SARS-CoV-2 ORF3a protein were synthesized by Synbio Technologies with an HA-tag at either the N-terminus (HA-ORF3a) or at the C-terminus (ORF3a-HA). Plasmids were sequenced to ensure that no deletions or other mutations were introduced during the synthesis. The expression of the ORF3a proteins was confirmed by transfection with the Turbofect transfection reagent (Thermo-Fisher, Waltham, MA, USA) into 293 cells for 48 h, followed by lysis of the cells in 1X RIPA and immunoblot analysis using a mouse monoclonal antibody directed against the HA-tag (Thermo-Fisher, catalog # 26183; antibody 2-2.2.14). Other plasmids that expressed organelle markers tagged with fluorescent proteins were used for the intracellular localization of ORF3a proteins. These included (1) ER-moxGFP for the rough endoplasmic reticulum (RER), Addgene catalog #68072 (a gift from Eric Snapp); (2) mNeonGreen-Giantin for cis-medial Golgi; Addgene catalog #98880 (a gift from Dorus Gadella); (3) TGN38 EGFP for the trans-Golgi network; Addgene catalog #128148 (a gift from Jennifer Lippincott-Schwartz); (4) 4xmts-mNeonGreen for mitochondria; Addgene catalog #98876 (a gift from Dorus Gadella); and (5) LAMP-1-mNeonGreen for lysosomes; Addgene #98882 (a gift from Dorus Gadella). Orf3a was detected in the LysoIP experiments using a rabbit monoclonal directed against ORF3a (Abcam, ab280953, Newark, DE, USA).

### 2.2. Site-Directed Mutagenesis of ORF3a

For site-directed mutagenesis, the pcDNA3.1(+) vector containing the SARS-CoV-2 HA-ORF3a gene was used in site-directed mutagenesis using a QuikChange II site-directed mutagenesis kit (Agilent, Santa Clara, CA, USA) according to the manufacturer’s protocol. A similar mutant was constructed using ORF3a with a C-terminal HA-tag. We found no differences in the intracellular localization of ORF3a with a C- or N-terminal HA-tag. For the construction of the ORF3a-[ΔYxxΦ], the tyrosine residues of the three potential tyrosine signals (^160^YNSV^163^, ^211^YYQL^214^, and ^233^YNKI^236^) were changed to alanine residues (^160^ANSV^163^, ^211^AYQL^214^, ^233^ANKI^236^). The ORF3a-[ΔYxxΦ] gene was sequenced to ensure that the desired changes were made and that no unwanted changes occurred during the mutagenesis process. Using ORF3a-[ΔYxxΦ] as a template, individual alanine residues were changed back to tyrosine residues to yield ORF3a-[Y160], ORF3a-[Y211], and ORF3a-[Y233]. ORF3a proteins with combinations of two tyrosine motifs were generated from the above single mutants to yield ORF3a-[Y160,211], ORF3a-[Y160,233], and ORF3a-[Y211,233]. Again, all *orf3a* genes were sequenced to ensure that the desired changes were made and that no unwanted changes occurred during the mutagenesis process.

### 2.3. Immunofluorescence Studies

To examine the intracellular localization of the SARS-CoV-2 ORF3a proteins, COS-7 cells grown on 13 mm coverslips were transfected with either the empty pcDNA3.1(+) vector, pcDNA3.1(+) expressing the unmodified ORF3a-HA, or the same vector expressing the various SARS-CoV-2 ORF3a mutants. The vectors were transfected into COS-7 cells using the Turbofect transfection reagent (ThermoFisher, Waltham, MA, USA) using the manufacturer’s instructions. With other experiments, vectors expressing unmodified HA-ORF3a or ORF3a mutants were co-transfected with vectors expressing various fluorescent marker proteins, as described above. At 48 h post-transfection, cells were washed three times in PBS, fixed in 4% paraformaldehyde (prepared in PBS) for 15 min, permeabilized with 0.1% Triton X-100 in PBS, and blocked for one hour with 22.5 mg/mL glycine and 0.1% BSA in PBST. The cultures were then incubated at 4C overnight with a mouse monoclonal antibody against HA-tag (Thermo-Fisher, antibody 2-2.2.14, #26183) and one of the following rabbit polyclonal or monoclonal antibodies: (a) ERGIC53 (ERGIC marker PTlabs; 13364-1-AP); (b) Golgin-97 (trans-Golgi marker; Abcam, ab84340) or (c) LAMP-1 (late endosome/lysosome marker; CST, D2D11). The cells were washed in PBS and incubated with a secondary goat anti-rabbit antibody conjugated to AlexaFluor™-488 (Invitrogen, Waltham, MA, USA, A11008) and a chicken anti-mouse conjugated to AlexaFluor™-594 (Invitrogen, A21201) for one hour. Cells were counter-stained with DAPI, and the coverslips were mounted on glass slides with ProLongTM Diamond Antifade Mountant (ThermoFisher, Waltham, MA, USA; catalog number P36961). The coverslips were viewed with a Leica TCS SP8 Confocal Microscope with a 100X objective and a 2X digital zoom using the Leica Application Suite X (LASX). A 405nm filter was used to visualize DAPI staining, a 488nm filter was used to visualize the organelle markers (RER, ERGIC, cis/medial Golgi, trans-Golgi, TGN, and late endosomes/lysosomes), and a 594 nm filter was used to visualize the HA-ORF3a protein. To examine the co-localization of the ORF3a proteins with mitochondria, COS-7 cells were co-transfected with vectors expressing the ORF3a proteins and a vector expressing 4xmts-NeonGreen (mitochondria; Addgene, #98876). At 48 h post-transfection, cells were fixed, permeabilized, and stained for HA-ORF3a proteins as described above. Micrographs of Z-stacks (0.7 μM each) were taken from the bottom to the top of the cell were taken from a minimum of 50 cells and the results presented in the figures were representative of each construct.

### 2.4. Surface/Internal Immunofluorescence Assays

To confirm the immunofluorescence results, we performed surface labeling experiments. COS-7 cells (2.5 × 10^5^) were plated onto cover slips in 35 mm dishes overnight. Cells were washed and transfected with 1.5 µg of plasmid expressing the HA-ORF3a or mutants. At 24 h, cells were fixed in 4% freshly prepared paraformaldehyde for 15 min and washed thrice with PBS, pH 7.4. The fixed cells were blocked with PBS containing 22.5 mg/mL glycine and 1% BSA for 1 h, washed, and incubated with the primary antibody (mouse anti-HA, 2-2.2.14 Invitrogen) in PBS containing 1% BSA at 1:400 dilution overnight at 4 °C. Cells were washed three times and incubated with the first secondary antibody (chicken anti-mouse-AF594, A21201, Invitrogen) in PBS containing 1% BSA 1 h at room temperature. Cells were permeabilized with PBS containing 0.2% Triton X-100 for 15 min and blocked with PBS containing 22.5 mg/mL glycine, 1% BSA, and 0.1% Tween-20. Cells were incubated with a 1:400 dilution of the primary antibody (mouse anti-HA, 2-2.2.14) in PBS containing 1% BSA and 0.1% Tween-20 for 1h at room temperature, washed three times, and incubated with a second secondary antibody (rabbit anti-mouse-AF488, A11059, Invitrogen) in PBS containing 1% BSA 1h at room temperature. Finally, cells were washed three times, stained with DAPI for 5 min, and mounted using Prolong Diamond Antifade Mountant (Invitrogen, P36961). Cells were examined as described above.

### 2.5. Isolation of Late Endosomes/Lysosomes from the Cells

We isolated lysosomes/early endosomes from cells transfected with the vectors expressing the unmodified ORF3a, the Orf3a-[ΔYxxΦ], or the empty pcDNA3.1(+) vector. We used the LysoIP protocol previously established by Abu-Remaileh and colleagues that isolates the late endosomes and lysosomal membranes based on the presence of transmembrane protein 192 (Tmem192) (Addgene, catalog #102930) [33]. As a negative control, we used pLJC-Tmem192-2XFlag (Addgene #102929). Retroviral stocks were prepared by transfecting HEK293 cells (80% confluency) with pLJC5-Tmem192-3XHA or pLJC-Tmem192-2XFlag (the negative control for the experiment) along with pVSV-G (envelope) and pCMV-dR8.2 dvpr (Gag-Pol and Rev) packaging plasmids. Cells were incubated at 37 °C for 48 h, virus-containing supernatants collected, centrifuged at 1000× *g* for 10 min, and frozen at −80 °C. Cell lines were established by expressing infecting HEK293 cells in 6-well plates with 8 µg/mL polybrene. At approximately 18 h post-infection, the medium was removed and replaced with a medium containing puromycin to select for cell lines expressing either Tmem192-3XHA or Tmem192-2XFlag. Stable cell lines were examined for proper intracellular localization by immunofluorescence using anti-HA or anti-Flag antibodies. To isolate late endosome/lysosomes, cell lines expressing Tmem192-3XHA or the Tmem192-2XFlag were transfected with either the empty pcDNA3.1(+) or vectors expressing ORF3a or ORF3a-[ΔYxxΦ] proteins (they were without an HA tag). At 48 h, cells were rinsed with PBS, scraped into 1.5 mL of KPBS (150 mM KCl, 10 mM K_2_PO_4_, pH 7.2), and centrifuged at 1000× *g* for 5 min. The pelleted cells were resuspended in 1.0 mL KPBS and an aliquot was reserved for the whole cell lysate fraction. The remaining lysate was homogenized with 20 strokes of a Dounce homogenizer and centrifuged at 1000× *g* for 10 min and resuspended with 175 µL of prewashed anti-HA magnetic beads prepared in KPBS. The beads were incubated for 15 min followed by collection of beads using a DynaMag spin magnet. The beads were washed three times in KPBS and were collected as described above. The membrane fraction was lysed in SDS-PAGE sample-reducing buffer, boiled and lysates separated using SDS-PAGE. The resolved proteins were transferred to PVDF membranes to be further analyzed by immunoblotting. Membranes were blocked with 5% nonfat dry milk prepared in TBST (Tris-buffered saline with Tween 20) for 1 h, then incubated overnight with primary antibodies in 5% bovine serum albumin (BSA) in TBST at 4 °C. The primary rabbit monoclonal antibodies (all from Abcam) were directed against LAMP-1 (ab208943), LAMP-2 (ab199946; both lysosomal membrane proteins), cathepsin C (CTSD) (ab75852; luminal lysosomal protein), calreticulin (ab2907; ER), S6K1 (ab131440; cytosol), and Giantin (ab317612; cis-medial Golgi) and ORF3a (ab280953). All primary antibodies were used at (1:1000) dilution. Following incubation, membranes were washed with TBST three times for 5 min and then incubated with the appropriate anti-rabbit secondary antibody diluted 1:2000 in 5% milk for 1 h at room temperature. Membranes were washed three times with TBST before being visualized using ECL Western blotting substrate.

### 2.6. Construction of the Infectious Clone of SARS-CoV-2 Delta

The infectious clone of the SARS-CoV-2 Delta variant (pSARS-CoV-2-Delta) was generated using transformation-associated recombination (TAR) cloning in *S. cerevisiae* strain VL6-48N [34]. The clone was assembled by recombining overlapping subgenomic fragments encompassing the entire SARS-CoV-2 genome with a bacterial/yeast artificial chromosome (BAC-YAC) vector2. The BAC-YAC vector enables the replication of the construct in both *S. cerevisiae* and *E. coli*.

Vero E6 cells were infected with SARS-CoV-2 Delta (B.1.617.2, Human, 2021, Germany ex India, 20A/452R, EVAg: 009V-04187) at a multiplicity of infection (MOI) of 1. Total RNA was extracted from the infected cells two days post-infection using TRIzol reagent (Thermo-Fisher Scientific). Complementary DNA (cDNA) synthesis was performed using SuperScript III reverse transcriptase (Thermo-Fisher). The complete SARS-CoV-2 genome was amplified as eleven overlapping fragments, each approximately 3 kb in length, with 64 to 346 bp overlaps between adjacent fragments to facilitate seamless assembly. High-fidelity VeriFi polymerase (PCR Biosystems, London, UK) was used to minimize unwanted mutations. The primers used to amplify the subgenomic fragments and the BAC-YAC vector are listed in Table 1.

The purified viral genome fragments and the BAC-YAC vector were assembled into the circular construct pSARS-CoV-2-Delta via TAR cloning [35]. Yeast colonies were selected on SD-His agar plates, and recombinant DNA was extracted from multiple colonies before being transferred into *E. coli* strain GS1783. Bacterial colonies were selected on LB agar plates supplemented with chloramphenicol (34 μg/mL). Plasmid DNA from several colonies was screened using restriction fragment length polymorphism (RFLP) analysis and full-genome sequencing via nanopore sequencing (Plasmidsaurus, South San Francisco, CA, USA). A construct with the expected sequence was designated as the parental clone for further mutagenesis.

### 2.7. Construction of Mutant BACs

To generate SARS-CoV-2 mutants carrying Y-to-A amino-acid substitutions in ORF3a, the desired mutations were introduced into the infectious BAC clone pSARS-CoV-2-Delta. The mutations involved exchanging the codons encoding tyrosine at positions Y160 (TAC), Y211 (TAT), or Y233 (TAC) with that of the codon GCA encoding alanine. In total, four mutant BAC constructs were engineered. One BAC construct carried all three Y-to-A mutations, designated as ORF3a-[ΔYxxΦ]. Three another BAC mutants carried only a single T-to-A mutation, designated as ORF3a-[Y160,211], ORF3a-[Y160,233], and ORF3a-[Y211,233]. In these designations, the numbers following Y indicate the positions of the intact tyrosine motifs within the ORF3a protein.

The mutations were introduced into pSARS-CoV-2-Delta using a two-step scarless *en passant*, a red-mediated recombination method in *E. coli* [36]. In the first step, the desired mutation was recombined into the target locus and selected using a kanamycin resistance gene. In the second step, the selection marker was cleaved by I-SceI digestion and subsequently removed through a second homologous recombination event, leaving only the intended mutation in the ORF3a gene. Kanamycin selection cassettes containing the mutations were amplified using VeriFi polymerase and primers listed in Table 2. 

The ORF3a-[ΔYxxΦ] construct was generated through three sequential rounds of mutagenesis. Following mutagenesis, mutant constructs were screened via RFLP analysis and verified by full-length genome sequencing using nanopore technology.

### 2.8. Rescue of Infectious SARS-CoV-2 Delta and Mutant ORF3a Viruses from BACs

High-quality BAC DNA was isolated from *E. coli* to recover infectious viruses using the Qiagen Midiprep kit. Infectious viruses were rescued from bacterial DNA in Vero E6 cells. To enhance recovery efficiency from Vero E6 cells, they were co-transfected with bacterial SARS-CoV-2 DNA and the dual-expression plasmid pVITRO2-EGFP-N, which expresses EGFP and the SARS-CoV-2 nucleocapsid protein under separate eukaryotic promoters [37]. Transfection was carried out using the Turbofect transfection reagent (Thermo-Fisher) following the manufacturer’s instructions. After a successful virus recovery, cell-culture supernatants containing the rescued viruses were collected for further characterization.

### 2.9. Virus Growth Curves

The genomes of all virus stocks were sequenced before infection experiments to confirm genetic integrity in most of the population, specifically at the furin cleavage site. Before experimental infection, virus stocks were stored at −80 °C. Growth curves were performed in Vero E6-TMPRSS2-T2A-ACE2 cells expressing human ACE-2 and TMPRSS2 (BEI Resources, Manassas, VA, USA). Vero E6 cells were grown on 60 mm dishes overnight at 37 °C. The next day, the medium was removed, and cells were inoculated with SARS-CoV-2 viruses at an M.O.I. of 0.1 in DMEM without serum for 2 h. The inoculum was removed and replaced with 3 mL of fresh DMEM containing 10% fetal bovine serum and antibiotics (5 µg/mL gentamicin plus 100 units/100 µg/mL of penicillin/streptomycin). Cells were incubated at 37C and medium removed (0.2 mL) at 0, 1, 2, 3, and 4 days post-inoculation. Ten-fold dilutions of the culture medium were prepared DMEM without serum, and titers were determined on Vero E6 cells. The cells were fixed and stained with methylene blue for 30 min. Plaques were counted for each dilution and titers calculated [38].

### 2.10. Pathogenicity with SARS-CoV-2 Viruses Expressing the Unmodified ORF3a or the ORF3a-[ΔYxxΦ] in K18hACE2 Mice

Six to eight week-old specific-pathogen-free female and male B6.Cg-Tg(K18-ACE2)2Prlmn/J (stock no.034860) K18 hACE2 transgenic mice were purchased from The Jackson Laboratory (Bar Harbor, ME, USA). K18 hACE2 transgenic mice were maintained in microisolator cages under ABSL3 conditions, provided sterile water and chow ad libitum, and acclimatized for 1 week before experimental manipulation. For morbidity and mortality studies, a total of 24 mice in three groups of eight animals (four female and four male). K18 hACE2 transgenic mice were either mock (DMEM)-infected or infected intranasally (i.n.) with 10^5^ PFU of the SARS-CoV-2 (WT) or SARS-CoV-2-(ORF3a-[ΔYxxΦ]) in a volume of 5 µL following isoflurane sedation. After viral infection, mice were monitored daily for 21 days for signs of morbidity (body-weight loss) and mortality (survival). Mice showing a >20% loss of their initial body weight were defined as reaching the experimental endpoint and were humanely euthanized. All mice were humanely euthanized at 21 days post-infection.

For the histological examination of tissues, aliquots of tissues were fixed in 10% neutral buffered formalin, tissues dehydrated in a series of alcohols (70%, 90%, 100%), and cleared to remove any alcohol. The tissues were embedded in paraffin wax, with 5 µm sections cut and mounted on glass slides, followed by hematoxylin and eosin (H&E) staining. Sections were viewed by light microscopy and scored for lesions by two veterinary pathologists.

## 3. Results

### 3.1. The ORF3a Has More than One Potential Tyrosine-Based Sorting Motif

**Figure 1:**
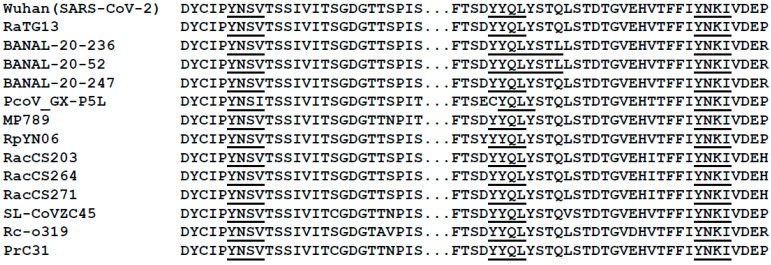
The alignment of SARS-CoV-2 and SARS-CoV-2-like ORF3a sequences (amino acids 155–240) with potential tyrosine-based sorting signals (YxxΦ) in the cytoplasmic domains is underlined and bolded. The isolate, species it was isolated from, and the NIH accession numbers are (1) Wuhan strain (Homo sapiens; accession #P0DTC3); (2) RATG13 (Rhinolophus affinis; accession #QHR63301); (3) Banal-20-236 (Rhinolophus marshalli; accession #UAY13254); (4) Banal- 20-52 (Rhinolophus marshalli; accession #UAY13218; (5) Banal-20-247 (Rhinolophus malayanus; accession #UAY13266); (6) PcoV_GX-P5L (Manis javanica; accession #QIA48633); (6) MP789 (Manis javanica) accession #QIG55946; (7) MP796 (Manis javanica, accession #QIG55946; (8) RpYN06 (Rhinolophus pusillus; accession #QWN56253); (9) RacCS203 (Rhinolophus acuminatus; accession #QQM18865); (10) RacCS264 (Rhinolophus acuminatus; accession #QQM18898); (11) RacCS271 (Rhinolophus acuminatus; accession #QQM18909); (12) SL-CoVZC45 (Rhinolophus pusillus; accession #AVP78032); (13) Rc-o319 (Rhinolophus cornutus; accession #BCG66628); and (14) PrC31 (Rhinolophus blythi; accession #QSQ01651). The potential tyrosine-based sorting signals are bolded and underlined.

We analyzed the ORF3a sequences from SARS-CoV-2 (274 amino acids in length) and SARS-CoV-2-like viruses (275 amino acids in length) for tyrosine-based sorting motifs (YxxΦ). The results indicated that three potential tyrosine-based sorting motifs were found in the cytoplasmic domain (Figure 1). All isolates had a conserved ^160^YNSV^163^ motif with two additional motifs detected at (^211^YYQL^213^ or ^211^YYQLYSTL^218^) and (^233^YNKI^236^), which were observed in all sequences (Figure 1). We also observed that the ORF3a proteins from SARS-CoV and SARS-CoV-like viruses also had two to four motifs (Supplementary Figure S1).

### 3.2. Expression of SARS-CoV-2 ORF3a and Various Mutants

**Figure 2:**
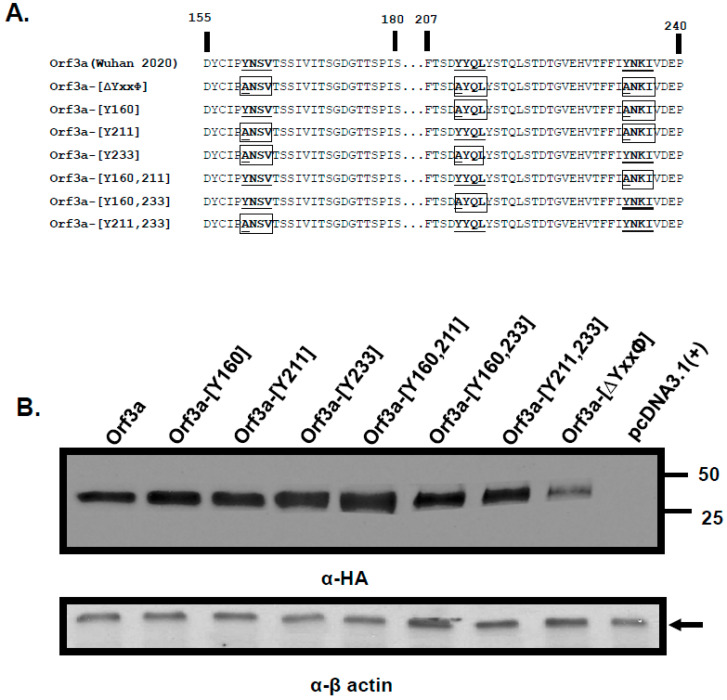
The ORF3a mutants analyzed in this study. (**A**). The unmodified ORF3a and the mutants were constructed for this study. The potential tyrosine-based sorting signals that were unchanged were bolded and underlined. Those potential tyrosine-based sorting signals that were altered are boxed and bolded with the amino-acid changes underlined. (**B**). The expression of the ORF3a and its mutants. HEK293 cells were transfected with vectors expressing the unmodified ORF3a, and the seven mutants were analyzed. Proteins were separated by SDS-PAGE, transferred to membranes, and analyzed in immunoblots using an antibody directed against the C-terminal HA-tag. β-actin served as a control for loading of samples.

We generated a series of ORF3a mutants in which one, two, or all three tyrosine residues of the three potential sorting motifs were changed to alanine residues. These mutants included a mutant in which all three tyrosines were altered to alanine (ORF3a-[ΔYxxΦ]), those mutants in which two tyrosine residues were substituted with alanines (ORF3a-[Y160], ORF3a-[Y211], and ORF3a-[Y233]), and those in which one tyrosine residue was substituted with alanines (ORF3a-[Y160,211], ORF3a-[Y160,233], and ORF3a-[Y211,233]). For the designation of the six mutants above, the number following the Y indicates the intact tyrosine motif(s). The unmodified ORF3a and mutants had an HA-tag at the N-terminus (Figure 2A). We examined the expression of ORF3a and the seven ORF3a mutants. The unmodified HA-ORF3a was stably expressed in 293 cells (Figure 2B). Our results indicated that all of the ORF3a mutants were expressed well save the ORF3a-[ΔYxxΦ], which was either expressed at lower levels or degraded faster than the other mutants.

### 3.3. The Unmodified SARS-CoV-2 ORF3a Protein Is Transported Through the Secretory Pathway to the Cell-Plasma Membrane

**Figure 3:**
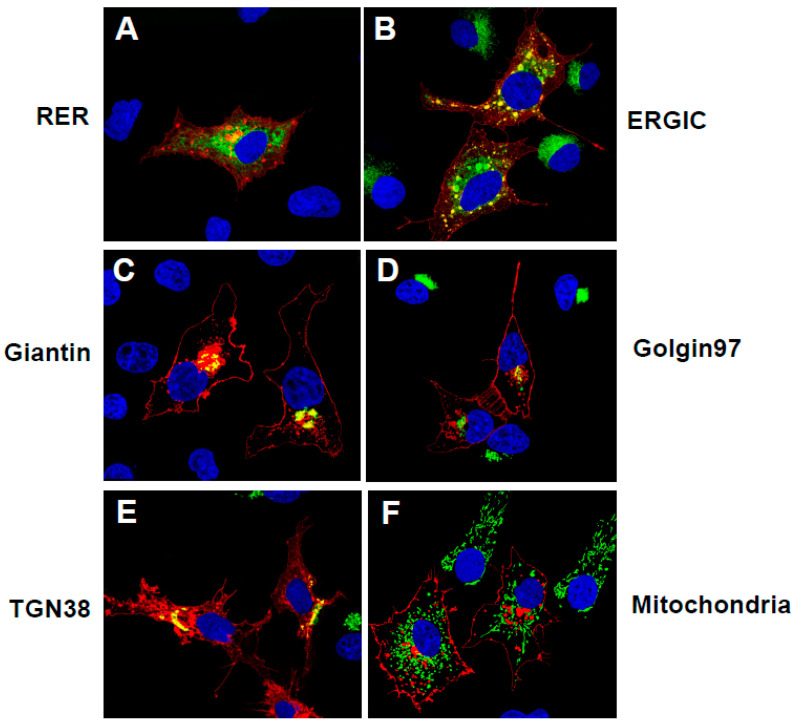
The SARS-CoV-2 ORF3a is expressed in organelles of the secretory pathway and at the cell-plasma membrane. COS-7 cells were co-transfected with the empty pcDNA.3.1(+) vector or vectors expressing SARS-CoV-2 ORF3a-HA protein and vectors expressing markers for the rough endoplasmic reticulum (RER; ERmoxGFP), cis/medial Golgi (mNeonGreen-Giantin), *trans* Golgi network (TGN38 EGFP), and mitochondria (4xmts-mNeonGreen). In other cultures, COS-7 cells were transfected with the pcDNA.3.1(+) vectors expressing SARS-CoV-2 HA-ORF3a and immunostained with antibodies against other intracellular organelles (ERGIC, or Golgin 97) as described in the Section 2. At 48 h post-transfection, cells were fixed, permeabilized, and blocked. The coverslips were reacted with a mouse monoclonal antibody against the HA-tag of HA-ORF3a and with a rabbit antibody against ERGIC53 (ERGIC) or Golgin 97 (*trans-*Golgi) followed by appropriate secondary antibodies, as described in the Section 2. Coverslips were washed, counter-stained with DAPI (1 μg/mL), and mounted. Cells were examined using a Leica TCS SPE confocal microscope, using a 100X objective with a 2X digital zoom and the Leica Application Suite X (LAS X) software package. A minimum of 100 cells were examined per staining, with the micrographs shown being representative. (**A**). Cells transfected with vectors expressing HA-ORF3a and ERmoxEGFP. (**B**). Cells transfected with a vector expressing HA-ORF3a and immunostained with antibodies against ERGIC-53 and HA. (**C**). Cells transfected with vectors expressing HA-ORF3a and mNeonGreen-Giantin. (**D**). Cells transfected with the vector expressing HA-ORF3a and immunostained with antibodies against Golgin 97. (**E**). Cells transfected with vectors expressing HA-ORF3a and TGN38 EGFP. (**F**). Cells transfected with the vector expressing HA-ORF3a and 4xmts-mNeon Green.

To investigate the intracellular trafficking of the unmodified SARS-CoV-2 ORF3a protein, cells were co-transfected with the vector expressing the ORF3a protein and vectors expressing each of the intracellular markers or immunostained with appropriate antibodies. With the co-transfections, cells were fixed at 48 h post-transfection and immunostained for ORF3a using an anti-HA antibody followed by a reaction with an appropriate secondary antibody. Our results indicate that the unmodified ORF3a co-localized with a marker for the ER, ERGIC, cis/medial Golgi, trans-Golgi, and the trans-Golgi network (TGN) and at the cell-plasma membrane (Figure 3).

### 3.4. The ORF3a-[ΔYxxΦ] Mutant Is Not Expressed at the Cell Surface

**Figure 4:**
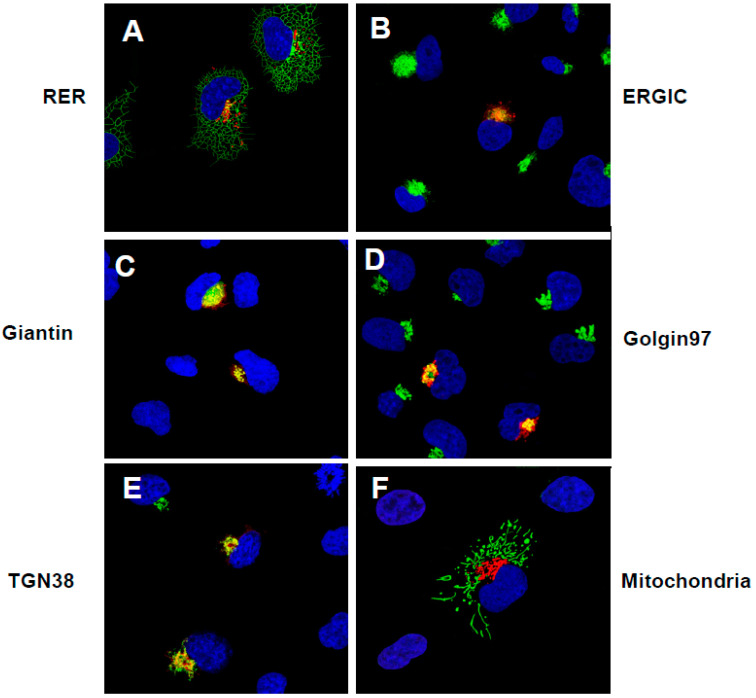
The ORF3a-ΔYxxΦ is not expressed at the cell-plasma membrane. HEK293 cells were transfected with the empty pcDNA.3.1(+) vector or a vector expressing the SARS-CoV-2 HA-ORF3a-[ΔYxxΦ] protein section as in Figure 3. (**A**). Cells transfected with vectors expressing HA-ORF3a-[ΔYxxΦ] and a vector expressing a marker for the rough endoplasmic reticulum (RER; ERmoxGFP) and immunostained with an anti-HA antibody. (**B**). Cells transfected with a vector expressing HA-ORF3a-[ΔYxxΦ] and immunostained with antibodies against ERGIC-53 and HA. (**C**). Cells transfected with vectors expressing HA-ORF3a-[ΔYxxΦ] and mNeonGreen-Giantin and immunostained with an anti-HA antibody. (**D**). Cells transfected with the vector expressing HA-ORF3a-[ΔYxxΦ] and immunostained with antibodies against Golgin 97 and HA. (**E**). Cells transfected with vectors expressing HA-ORF3a-[ΔYxxΦ] and TGN38 EGFP and immunostained with an anti-HA antibody. (**F**). Cells transfected with the vector expressing HA-ORF3a-[ΔYxxΦ] and 4xmts-mNeonGreen and immunostained with antibodies against HA.

Next, we analyzed ORF3a-[ΔYxxΦ] for its intracellular localization with appropriate intracellular markers, as had been carried out for the unmodified ORF3a. We observed that the ORF3a protein co-localized with markers for the ER, ERGIC, *cis*-*medial* Golgi, *trans-*Golgi, and the TGN. Unlike the unmodified ORF3a, ORF3a-[ΔYxxΦ] was not detected at the cell-plasma membrane of transfected cells (Figure 4). None of the ORF3a constructs co-localized with a marker for mitochondria.

### 3.5. Expression of ORF3a Mutants with One or Two Potential Tyrosine-Based Sorting Motifs Intact

**Figure 5:**
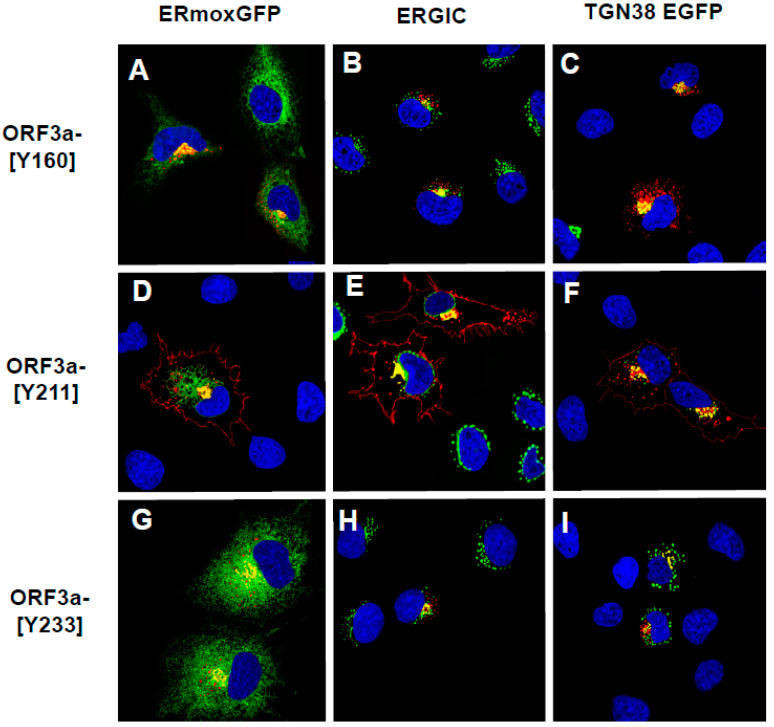
Cell expression of ORF3a mutants with one tyrosine-based motif intact. COS-7 cells were co-transfected with vectors expressing HA-ORF3a, HA-ORF3a-[Y160], HA-ORF3a-[Y211], or HA-ORF3a-[Y233] and a vector expressing ER-moxEGFP or TGN38-EGFP. At 48 h post-transfection, cells were fixed, permeabilized, and blocked. Cells were reacted with a mouse monoclonal antibody against the HA-tag overnight, and washed and reacted with an appropriate secondary antibody tagged with Alexa Fluor 594 (for HA) for 1 h. Cells were washed and counter-stained with DAPI (1 μg/mL) for 5 min. Cells were viewed using a Leica TC8 confocal microscope as described in the Section 2. At least 50 cells were examined for surface expression and co-localization with ERmoxGFP or TGN38 EGFP. (**A**). Cells transfected with a vector expressing HA-ORF3a-[Y160] and ERmoxGFP and immunostained with antibodies against the HA-tag. (**B**). Cells transfected with a vector expressing HA-ORF3a-[Y160] and immunostained with antibodies against the HA-tag and ERGIC-53. (**C**). Cells transfected with a vectors expressing HA-ORF3a-[Y160] and TGN38 EGFP and immunostained with an antibody against the HA-tag. (**D**). Cells transfected with vectors expressing HA-ORF3a-[Y211] and ERmoxGFP and immunostained with antibodies against the HA-tag. (**E**). Cells transfected with a vector expressing HA-ORF3a-[Y160] and immunostained with antibodies against the HA-tag and ERGIC-53. (**F**). Cells transfected with a vector expressing HA-ORF3a-Y211 and TGN38 EGFP and immunostained with antibodies against the HA-tag. (**G**). Cells transfected with vectors expressing HA-ORF3a-[Y233] and ERmoxGFP and immunostained with antibodies against the HA-tag. (**H**). Cells transfected with vectors expressing HA-ORF3a-[Y233] and immunostained with antibodies against the HA-tag and ERGIC-53. (**I**). Cells transfected with a vector expressing HA-ORF3a-[Y160] and TGN38 EGFP and immunostained with antibodies against the HA-tag.

We next examined the intracellular localization of ORF3a mutants with one potential tyrosine-based motif intact (ORF3a-[Y160], ORF3a-[Y211], and ORF3a-[Y233]). (Figure 5A–C). The co-transfection of vectors expressing the ORF3a-[Y160] (Figure 5A) or ORF3a-[Y233] (Figure 5C) with vectors expressing the ERmoxGFP fusion protein revealed that both proteins co-localized with the ERmoxGFP fusion protein but were not observed at the cell-plasma membrane (Figure 5A–C,G–I). The transfection of cells with the vector expressing ORF3a-[Y211] revealed that this protein co-localized with the ERmoxGFP marker and was readily detectable at the cell surface (Figure 5D–F).

**Figure 6:**
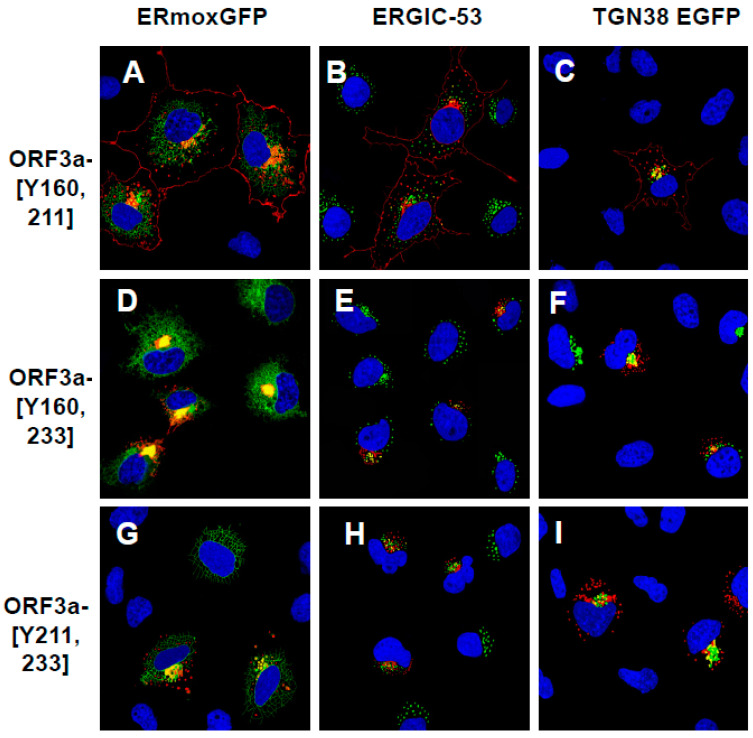
The cell expression of ORF3a mutants with two intact tyrosine motifs intact. COS-7 cells were co-transfected with vectors expressing HA-ORF3a-[Y160,211] (**A**–**C**), HA-ORF3a-[Y160,233] (**D**–**F**), or HA-ORF3a-[Y211,233] (**G**–**I**) and ERmoxGFP (**A**,**D**,**G**) or TGN38-GFP (**C**,**F**,**I**). Cells were also transfected with the ORF3a mutants above and immunostained for ERGIC-53 (**B**,**E**,**H**). At 48 h post-transfection, cells were fixed, permeabilized, and blocked. Cells were reacted with a mouse monoclonal antibody against the HA-tag overnight, washed, and reacted with an appropriate secondary antibody tagged with Alexa Fluor 594 for HA for 1 h. Cells were washed and counter-stained with DAPI (1 μg/mL) for 5 min. Cells were viewed using a Leica TC8 confocal microscope as described in the Section 2. At least 50 cells were examined for expression and co-localization. (**A**–**C**). Cells transfected with vectors expressing HA-ORF3a-[Y160,211] and ERmoxGFP (**A**), transfected with HA-ORF3a-[Y160,211] and immunostained for ERGIC-53 and HA (**B**) or transfected with vectors expressing HA-ORF3a-[Y160,211] and TGN38 EGFP and immunostained for HA (**C**). ERGIC-53 and immunostained with antibodies against the HA-tag. (**D**–**F**). Cells transfected with vectors expressing HA-ORF3a-[Y160,233] and ERmoxGFP (**D**), transfected with a vector expressing HA-ORF3a-[Y160,233] and immunostained for ERGIC-53 (**E**) or transfected with vectors expressing HA-ORF3a-[Y160,233] and TGN38 EGFP (**F**). (**G**–**I**). Cells transfected with vectors expressing HA-ORF3a-[Y211,233] and ERmoxGFP (**G**), transfected with HA-ORF3a-[Y211,233] and immunostained for ERGIC-53 (**H**) or transfected with vectors expressing HA-ORF3a-[Y211,233] and TGN38 EGFP (**I**).

We also analyzed the ORF3a mutants with two intact tyrosine motifs (ORF3a-[Y160,211]; ORF3a-[Y160,233]; and ORF3a-[Y211,233]) (Figure 6). The rationale behind analyzing these mutants was to determine if more than one tyrosine motif was required for efficient trafficking to the cell surface. Our results indicated that of these three mutants, only ORF3a-[Y160,211] colocalized with the ERmoxGFP, ERGIC-53, and TGN-38-GFP and was expressed at the cell surface (Figure 6A–C). In contrast, ORF3a-[Y160,233] and ORF3a-]Y211,233] colocalized with the ERmoxGFP, ERGIC-53, and TGN-38-GFP markers but were not detected at the cell surface (Figure 6D–I).

### 3.6. The Surface Immunostaining of Cells Transfected with Vectors Expressing the ORF3a and ORF3a Mutants Confirms the Cell Surface Expression Patterns

To confirm that ORF3a, ORF3a-[Y211], and ORF3a-[Y160,211] were expressed at the cell surface, we also performed a double immunostaining assay, relying on sequential treatment with antibodies before and after cell permeabilization, as described in the Materials and Methods. Both immunostainings were performed on ice. If the ORF3a or various mutants were transported to the cell surface, the plasma membrane should stain with the Alexa Fluor 594 while internal ORF3a proteins should stain with the Alexa Fluor 488. Conversely, if the ORF3a protein was not expressed on the cell surface, immunostaining with the anti-HA and the Alexa Fluor 594 should yield little to no red signal, while intracellular ORF3a should still be detected with anti-HA and Alexa Fluor 488 staining.

All micrographs were taken using the same exposure time and laser intensity.

**Figure 7:**
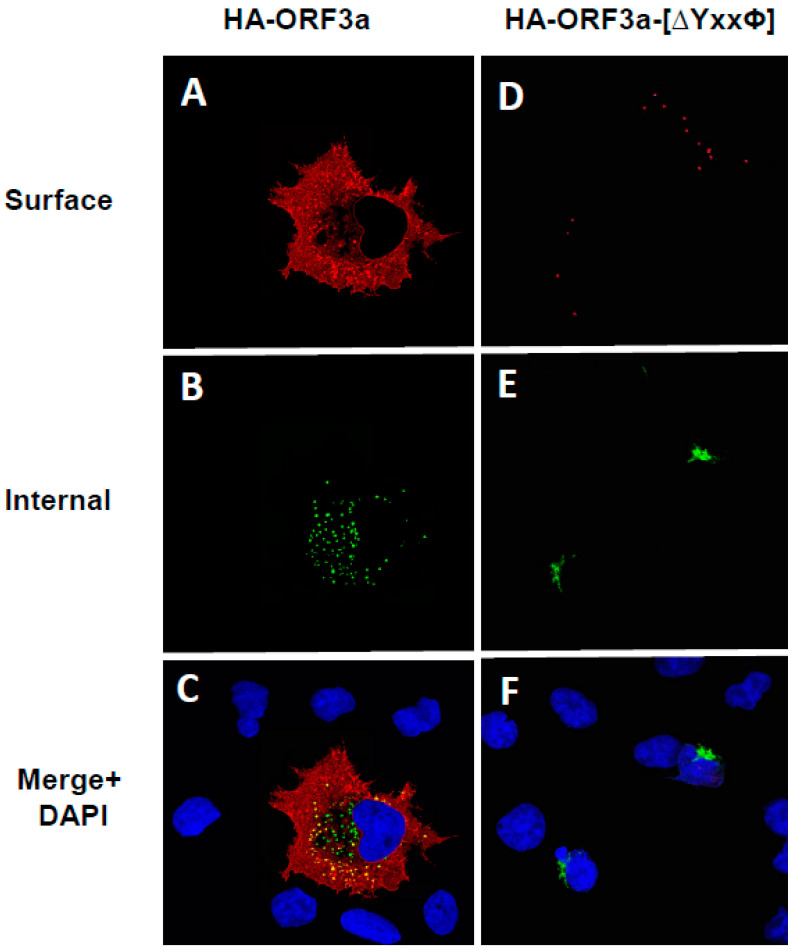
Surface immunostaining of cells transfected with vectors expressing ORF3a and ORF3a-YxxP. COS-7 cells were transfected with vectors expressing either ORF3a (**A**–**C**) or ORF3a-YxxP (**D**–**F**). COS-7 cells transfected with the empty vector showed no immunofluorescence. At 24 h post-transfection, cells were immunostained with an antibody against the HA-tag followed by a secondary antibody tagged with Alexa Fluor 594. Cells were washed three times and permeabilized as described in the Section 2. Permeabilized cells were then reacted with the same primary antibody and a secondary antibody tagged with Alexa Fluor 488. The cells were washed, mounted, and examined using a Leica TSP8 laser-scanning confocal microscope. (**A**). The surface immunostaining of ORF3a with an antibody against the HA-tag. (**B**). The internal immunostaining of ORF3a with an antibody against the HA-tag. (**C**). Merge of (**A**,**B**). (**D**). The surface immunostaining of ORF3a-[YxxΦ] with an antibody against the HA-tag. (**E**). The internal immunostaining of ORF3a-[YxxΦ] with an antibody against the HA-tag. (**F**). The merging of (**D**,**E**).

**Figure 8:**
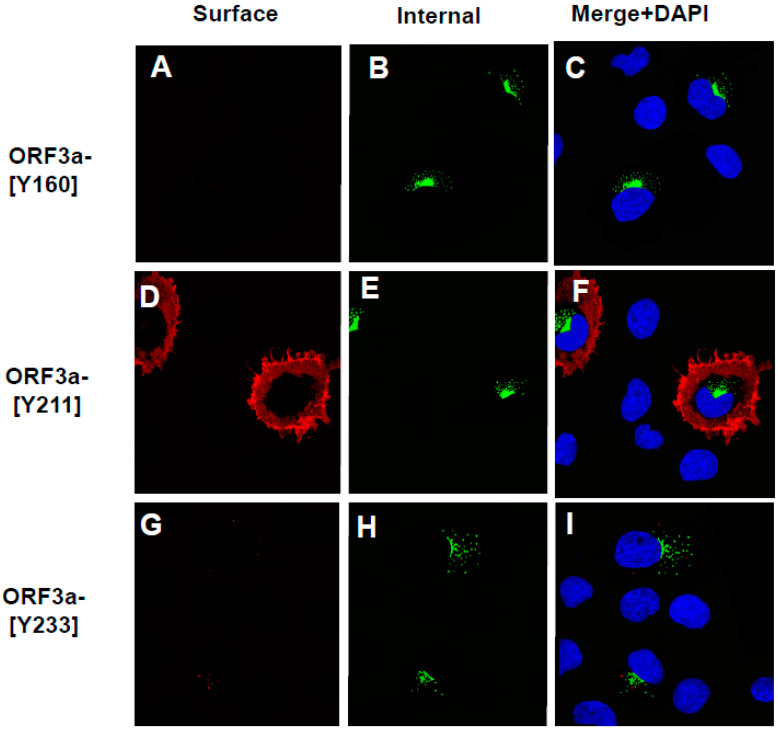
The surface immunostaining of cells transfected with vectors expressing ORF3a mutants with one tyrosine motif intact. COS-7 cells were transfected with vectors expressing each of the ORF3a mutants. At 24 h post-transfection, cells were immunostained with an antibody against the HA-tag followed by a secondary antibody tagged with Alexa Fluor 594. The cells were washed three times, and permeabilized as described in the Section 2. Permeabilized cells were then reacted with the same primary antibody and a secondary antibody tagged with Alexa Fluor 488. The cells were washed, mounted, and examined using a Leica TSP8 laser-scanning confocal microscope. Shown are individual red and green images and merged images of the red, green, and blue channels. (**A**). The surface immunostaining of HA-ORF3a-[Y160] with an antibody against the HA-tag. (**B**). The internal immunostaining of HA-ORF3a-[Y160] with an antibody against the HA-tag. (**C**). The merging of (**A**,**B**). (**D**). The surface immunostaining of HA-ORF3a-[Y211] with an antibody against the HA-tag. (**E**). The internal immunostaining of HA-ORF3a-[Y211] with an antibody against the HA-tag. (**F**). The merging of (**D**,**E**). (**G**). The surface immunostaining of HA-ORF3a-[Y233] with an antibody against the HA-tag (**H**). The internal immunostaining of HA-ORF3a-[Y233] with an antibody against the HA-tag (**I**). The merging of cells (**G**,**H**).

**Figure 9:**
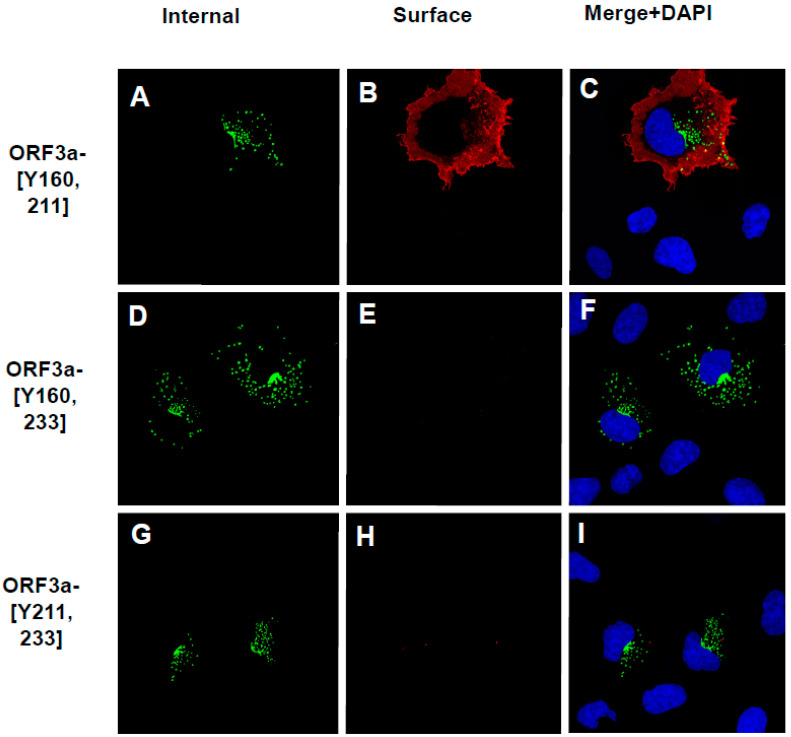
The Surface immunostaining of cells transfected with vectors expressing ORF3a mutants with two tyrosine motifs intact. COS-7 cells were transfected with vectors expressing each of the ORF3a mutants. At 24 h post-transfection, cells were immunostained with an antibody against the HA-tag followed by a secondary antibody tagged with Alexa Fluor 594. The cells were washed three times and permeabilized as described in the Section 2. Permeabilized cells were then reacted with the same primary antibody and a secondary antibody tagged with Alexa Fluor 488. Cells were washed, mounted, and examined using a Leica TSP8 laser-scanning confocal microscope. Individual red and green images and merged images of the red, green, and blue channels are shown. (**A**). The internal immunostaining of HA-ORF3a-[Y160,211] with an antibody against the HA-tag. (**B**). The surface immunostaining of HA-ORF3a-[Y160,211] with an antibody against the HA-tag. (**C**). The merging of (**A**,**B**). (**D**). The internal immunostaining of HA-ORF3a-[Y160,233] with an antibody against the HA-tag. (**E**). The surface immunostaining of HA-ORF3a-[Y160,233] with an antibody against the HA-tag. (**F**). The merging of (**D**,**E**). (**G**). The internal immunostaining of HA-ORF3a-[Y211,233] with an antibody against the HA-tag (**H**). The surface immunostaining of HA-ORF3a-[Y211,233] with an antibody against the HA-tag. (**I**). The merging of cells (**G**,**H**).

As expected, cells transfected with the vector expressing the unmodified HA-ORF3a were stained at the cell surface (evidenced by the red color) (Figure 7A). In contrast, cells transfected with the vector expressing HA-ORF3a-[ΔYxxΦ] revealed minimal immunostaining at the cell surface (Figure 7D). Following permeabilization and immunostaining for internal ORF3a, both constructs exhibited intracellular staining (Figure 7B,E), and merged images taken at 488nm and 594nm confirmed the surface staining of HA-ORF3a but not HA-ORF3a-[ΔYxxΦ] (Figure 7C,F). Both constructs exhibited intracellular staining (Figure 7B,E), and merged images taken at 488nm and 594nm confirmed the surface staining of HA-ORF3a but not HA-ORF3a-[ΔYxxΦ] (Figure 7C,F). Using the same methodology, mutants HA-ORF3a-[Y160], HA-ORF3a-[Y233], HA-ORF3a-[Y160,233], and HA-ORF3a-[Y211,233] were not detected on the cell surface, while both HA-ORF3a-[Y211] and HA-ORF3a-[Y160,211] were clearly expressed at the cell surface (Figure 8 and Figure 9). Taken together, these results indicate that (a) the tyrosine motif ^160^YNSV^163^ alone is insufficient for transport of ORF3a to the cell surface; (b) the tyrosine motif ^211^YYQL^214^ alone can target ORF3a to the cell surface; (c) the combination of tyrosine motifs ^160^YNSV^163^ and ^211^YYQL^214^ facilitates ORF3a transport to the cell-plasma membrane; and d) ORF3a mutants retaining only the tyrosine motif ^160^YNSV^163^ (Orf3a-[Y160]) or the ^233^YNKI^236^ (ORF3a-[Y233]) were not detected at the cell surface.

### 3.7. Substitution of the Tyrosine Residues in the Three Motifs with Phenylalanine Residues

**Figure 10:**
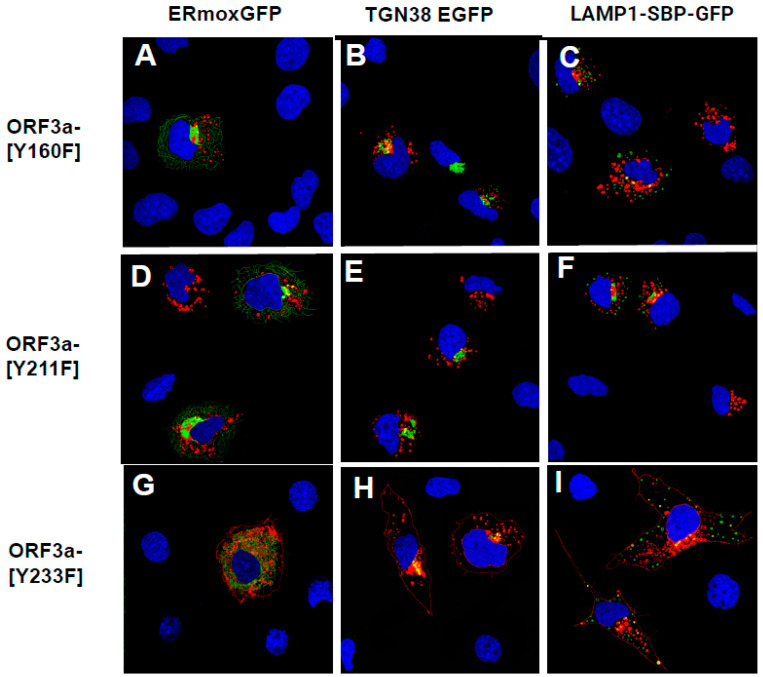
Phenylalanine residues cannot substitute for tyrosine residues in the tyrosine motifs. COS-7 cells were grown in 6-well plates with coverslips. Cells (70% confluent) were co-transfected with vectors expressing HA-ORF3a-[Y160F] (**A**–**C**), HA-ORF3a-[Y211F] (**D**–**F**), or HA-ORF3a-[Y233F] (**G**–**I**) and either ERmoxGFP (**A**,**D**,**G**), TGN-38GFP (**B**,**E**,**H**), or LAMP-1-GFP (**C**,**F**,**I**). At 48 h post-transfection, the cells were processed for immunofluorescence as described in Figure 3.

A potential caveat is that substituting the tyrosine residues with alanines may have altered the structure of the cytoplasmic domain resulting in the observed results. To address this potential concern, we substituted the tyrosine residues of the three potential tyrosine motifs with structurally similar phenylalanine residues. Previously, it was shown that tyrosine could not be effectively substituted by the phenylalanine residues (which are structurally similar) in a sorting motif [38]. These three constructs, designated as HA-ORF3a-[Y160F], HA-ORF3a-[Y211F], and HA-ORF3a-[Y233F] (with the other two tyrosine motifs intact), were analyzed for ORF3a expression in the RER, TGN, late endosomes/lysosomes and at the cell-plasma membrane. The results indicate that HA-ORF3a-[Y211F] and HA-ORF3a-[Y160F] were only detected intracellularly, whereas HA-ORF3a-[Y233F] was detected at the cell surface (Figure 10).

### 3.8. Lysosome Localization of the ORF3a Mutants

Tyrosine-based sorting signals are important for targeting proteins to lysosomes [21,22,23]. To investigate whether ORF3a follows this trafficking route, we then analyzed the unmodified ORF3a, and ORF3a mutants for co-expression with lysosome-associated membrane protein 1 (LAMP-1), a marker associated with late endosomes and lysosomes. Vectors expressing the unmodified HA-ORF3a or each mutant were transfected into COS-7 cells. At 48 h post-transfection, the cells were fixed and permeabilized followed by immunostaining for the HA-tag on the ORF3a protein and for LAMP-1. The cells were examined using laser-scanning confocal microscopy for LAMP-1 and ORF3a proteins.

**Figure 11:**
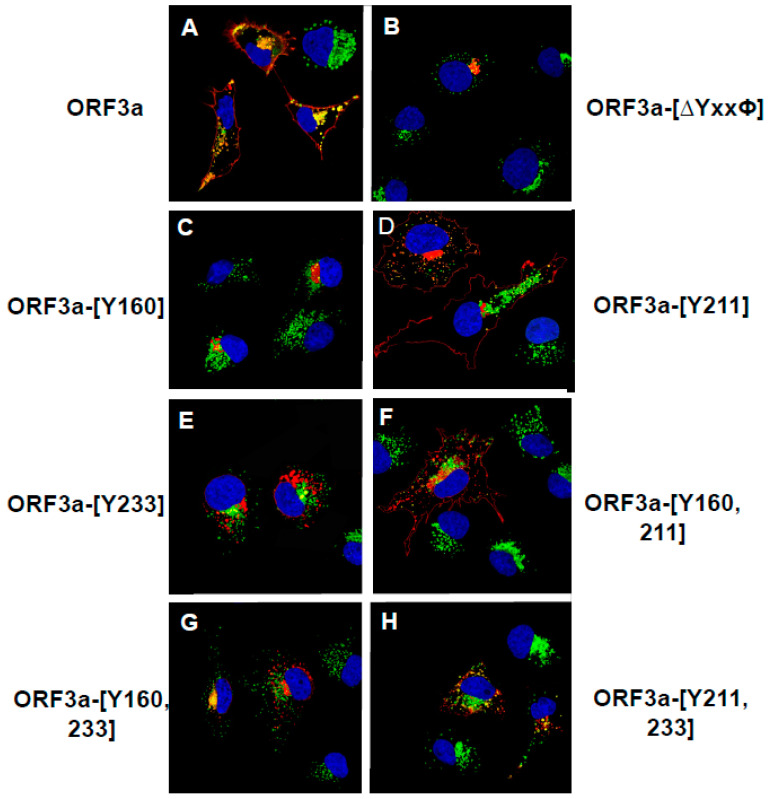
The co-localization of ORF3a and the tyrosine-based sorting signal mutants with LAMP-1. COS-7 cells were transfected with the empty vector or the same vector expressing ORF3a or individual ORF3a mutants. At 48 h post-transfection, the cells were fixed, permeabilized, and blocked. Cells were reacted with a mouse monoclonal antibody against the HA-tag and a rabbit antibody against LAMP-1 overnight, washed, and reacted with an appropriate secondary antibody tagged with Alexa Fluor 594 (for HA) and Alexa Fluor 488 (for LAMP-1) for 1 h. Cells were washed and counter-stained with DAPI (1 μg/mL) for 5 min. Cells were viewed using a Leica TC8 confocal microscope and at least 50 cells were examined for co-localization with the LAMP-1 marker. (**A**). HA-ORF3a. (**B**). HA-ORF3a-[YxxΦ]. (**C**). HA-ORF3a-[Y160]. (**D**). HA-ORF3a-[Y211]. (**E**). HA-ORF3a-[Y233]. (**F**). HA-ORF3a-[Y160,211]. (**G**). HA-ORF3a-[Y160, 233]. (**H**). HA-ORF3a-[Y211,233].

**Figure 12:**
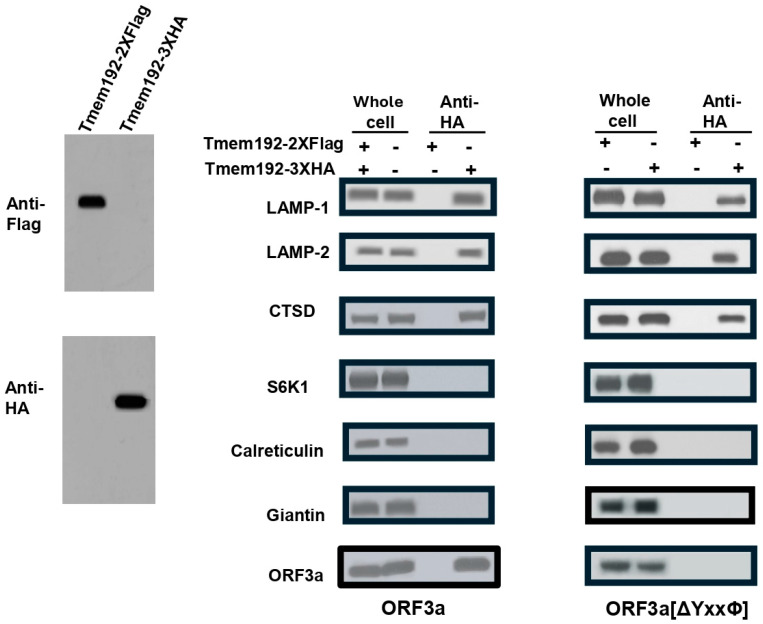
Orf3a but not ORF3a-[ΔYxxΦ] co-purifies with lysosomes/late endosomes. Retroviral stocks were prepared by transfecting HEK293 cells with pLJC5-Tmem192-3XHA or pLJC-TMEM192-2XFlag (the negative control for the experiment) along with pVSV-G (envelope) and pCMV-dR8.2 dvpr (Gag-Pol and Rev) packaging plasmids and cell lines established. Next, these cell lines were transfected with vectors expressing ORF3a (left panel) or ORF3a-[ΔYxxΦ] proteins (right panel). Late endosomes/lysosomes were purified using the LysoIP technique described in the Section 2. Samples were analyzed by immunoblots for the presence of lysosome markers (LAMP-1, LAMP-2, cathepsin D [CTSD]), cytosol (SK61), rough endoplasmic reticulum (calreticulin), and ORF3a using appropriate antibodies. Following incubation, membranes were washed and incubated with the appropriate anti-rabbit secondary antibodies, and membranes were washed three times with TBST before being visualized using ECL substrate.

The results revealed that the unmodified ORF3a co-localized with LAMP-1 in both the region of the RER, and in vesicular structures closer to the cell-plasma membrane (Figure 11). In cells expressing HA-ORF3a-[ΔYxxΦ], HA-ORF3a-[Y160], HA-ORF3a-[211], HA-ORF3a-[Y233], HA-ORF3a-[Y160,211], HA-ORF3a-[Y211,233], or HA-ORF3a-[Y160,233], these proteins co-localized with LAMP-1 in the RER region, but only HA-ORF3a-[Y211] and HA-ORF3a-[Y160,211] also co-localized with LAMP-1 positive lysosomes towards the periphery of the cell (Figure 12). This suggests that targeting ORF3a to the lysosomes required the tyrosine motif ^211^YYQL^214^.

### 3.9. Purification of Late Endosomes/Lysosomes Reveals That ORF3a-[ΔYxxФ] Does Not Traffic to These Compartments of the Cell

As the above immunofluorescence studies showed co-localization of ORF3a but not ORF3a-[ΔYxxФ] with the LAMP-1 marker, we used a late endosome/lysosome isolation technique to determine if ORF3a and ORF3a-[ΔYxxФ] trafficked to the late endosome/lysosome compartments. Using a previously described LysoIP technique, we determined if ORF3a and ORF3a-[ΔYxxФ] partitioned into lysosomal fractions. HEK293 cell lines stably expressing pLJC5-TMEM192-3XHA or pLJC-TMEM192-2XFlag (used as negative control) were transfected with vectors expressing HA-ORF3a or HA-ORF3a-[ΔYxxФ] and at 48 h the post-transfection membranes were isolated. The collected fractions were analyzed for the presence of lysosome, cytosol, RER, Golgi complex, and ORF3a proteins.

The results indicate that the TMEM192-3XHA or TMEM192-2XFlag proteins were expressed in whole-cell lysates. In the LysoIP fractions, ORF3a protein was detectable in the TMEM192-3XHA but not in the TMEM192-2XFlag fractions, confirming specific lysosomal localization. However, ORF3a-[ΔYxxФ] protein was undetectable in TMEM192-3XHA or TMEM192-2XFlag fractions. While we acknowledge that the HA-ORF3a-[ΔYxxΦ] mutant is likely not expressed at the same level or is less stable than HA-ORF3a, these results confirm that while HA-ORF3a but not HA-ORF3a-[ΔYxxФ] is present in the late endosome/lysosome compartments, reinforcing the role of tyrosine-based sorting signals in lysosomal targeting but not necessarily in virus release (see Section 4).

### 3.10. Replication of SARS-CoV-2 and SARS-CoV-2 Mutants in Vero E6 Cells

**Figure 13:**
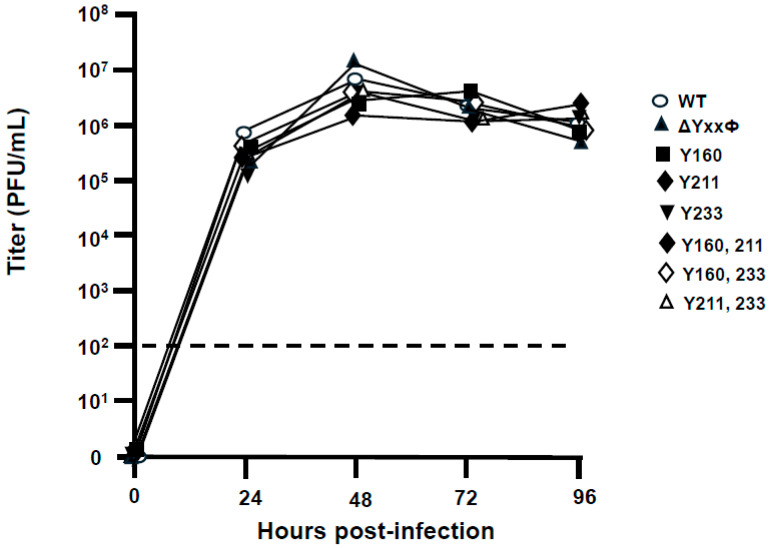
The replication of the unmodified SARS-CoV-2 (WT) and the viruses expressing the mutant ORF3a proteins in VeroE6 cells. VeroE6 cells expressing the hACE-2 receptor were seeded into six-well plates and allowed to grow for 24 h. Viruses that were diluted (those unmodified and those expressing the mutant ORF3a proteins) and cells inoculated with the virus at an M.O.I. of 0.01 in 0.5 mL of DMEM for two hours. The inoculum was removed, fresh medium (DMEM90FBS10) was added, and incubated for 4 days at 37C. Aliquots of the medium were collected at 0, 1, 2, 3, and 4 days post-infection. The medium was clarified by low-speed centrifugation, and the infectious virus was titered on VeroE6 cells in 6-well plates. Cells were fixed with 10% formaldehyde and stained with methylene blue to visualize plaques.

The replication of wild-type and ORF3a mutants was determined in Vero E6 cells. Growth curves showed that the unmodified SARS-CoV-2 and SARS-CoV-2 mutant viruses replicated similarly in Vero E6 cells [Figure 13]. These results are similar to those from a previous study that showed a SARS-CoV-2 virus with the *orf3a* gene deleted replicated to similar titers in this cell line [38,39].

### 3.11. A SARS-CoV-2 Virus with All Three Tyrosine Motifs Removed (SARS-CoV-2-[ORF3a-[ΔYxxΦ]) Was Less Pathogenic in K18-hACE2 Mice

As the ORF3a-[ΔYxxΦ] was not detected at the cell surface of cells, we investigated whether a SARS-CoV-2 virus expressing the mutant ORF3a lacking all three potential tyrosine-based sorting signals (Orf3a-[ΔYxxΦ]) exhibited altered pathogenicity in vivo. For these experiments, K18-hACE2 mice in three groups (8 mice per group) were inoculated intranasally with 20 µL of either DMEM (mock control), the virus expressing the unmodified ORF3a protein (SARS-CoV-2; 10^5^ TCID_50_), or the virus expressing the ORF3a-[ΔYxxΦ] the (SARS-CoV-2-[ORF3a-ΔYxxΦ]); 10^5^ TCID_50_). The mice were monitored daily for 21 days for clinical signs of morbidity (weight loss) and mortality (survival).

**Figure 14:**
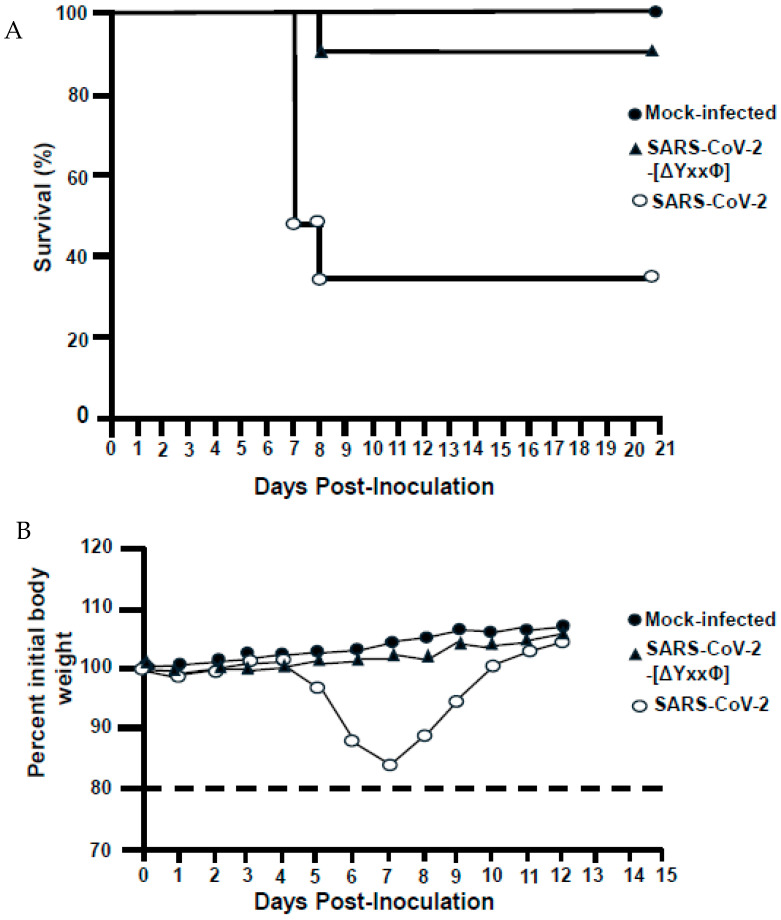
The SARS-CoV-2-[ORF3a-ΔYxxΦ] was less pathogenic in K18-hACE2 mice. (**A**). K18-hACE2 mice in three groups (eight per group) were inoculated with (i) DMEM (vehicle control), (ii) 5 × 10^4^ TCID_50_ of SARS-CoV-2 expressing the unmodified ORF3a, or (iii) 5 × 10^4^ TCID_50_ of a SARS-CoV-2 virus expressing the ORF3a-YxxΦ. The survival of K18hACE-2 mice was evaluated at the indicated times post-infection. (**B**) The mean body weight of mice infected from the three groups. Mice were followed for 21 days, with weights determined daily and when unhealthy mice were euthanized. All mice were euthanized at 21 days post-infection. The survival analysis of the mice following inoculations revealed that all mice inoculated with vehicle control (DMEM) remained healthy through the 21-day experiment (**B**). Five of eight mice infected with SARS-CoV-2 with the unmodified ORF3a were moribund and were euthanized on days 7 (4) and 8 (1). Three of eight mice remained healthy through 21 days. Finally, only one of eight mice infected with SARS-CoV-2-[ORF3a-ΔYxxΦ] was euthanized at 8 days post-infection in a moribund condition, while the other seven mice showed no clinical signs of disease in the study (**B**).

Mice inoculated with DMEM showed no significant weight loss throughout the 21-day experiment (Figure 14B). In contrast, mice infected with the unmodified SARS-CoV-2 virus lost weight from day 6 to 9. Finally, all but one mouse infected with SARS-CoV-2-[ORF3a-ΔYxxΦ] did not lose significant weight (most were above the initial weight at day 0) (see Supplementary Figure S2 for the individual weights).

**Figure 15:**
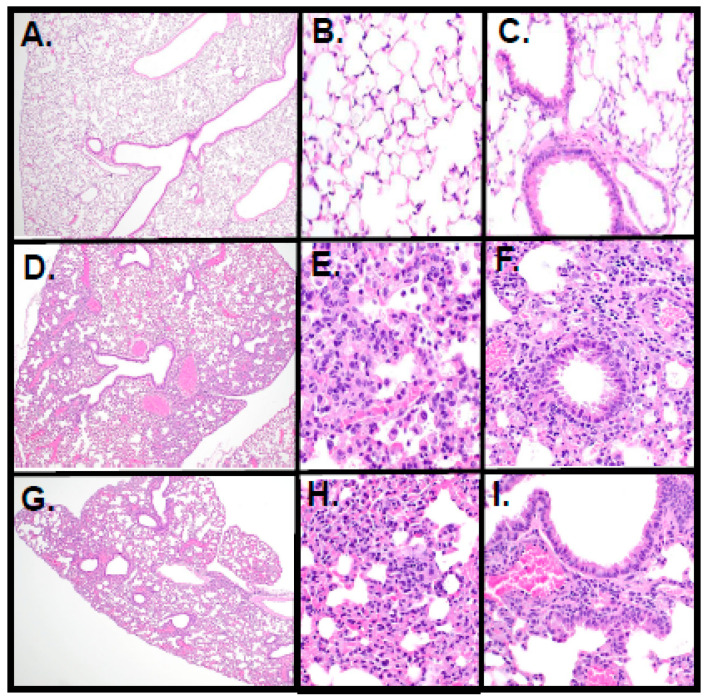
Lung histopathology of vehicle control, SARS-CoV-2-infected, and SARS-CoV-2- or Orf3a[YxxΦ]-infected mice. (**A**–**C**). Vehicle control at 21 days post-infection. No lesions or rare mononuclear cells in alveolar spaces of the adventitia of bronchi or vasculature. (**D**–**F**). SARS-CoV-2 (7 days post-infection). (**D**). Moderate, multifocal to coalescing, non-suppurative, broncho-interstitial pneumonia with alveolitis. (**E**). The consolidation of alveolar spaces, thickening of alveolar septa, and cellular infiltrates within alveolar spaces. (**F**). Moderate hyperplasia, mild dysplasia of the bronchial epithelium, and mild to moderate non-suppurative peribronchial and perivascular inflammation. (**G**–**I**). SARS-CoV-2-ORF3a-[YxxΦ] (8 days post-infection). (**G**). Mild to moderate, multifocal, non-suppurative, broncho-interstitial pneumonia. (**H**). The consolidation of alveolar spaces, thickening of alveolar septa, and cellular debris filling alveolar spaces. (**I**). Moderate hyperplasia and mild dysplasia of bronchiolar and terminal bronchiolar epithelium with mild segmental non-suppurative peribronchial and perivascular inflammation.

Lung tissues were examined histologically from the mice of the three groups. As expected, mice inoculated with DMEM did not show lesions or rare mononuclear cells in alveolar spaces (Figure 15A–C). In contrast, mice that succumbed to SARS-CoV-2 infection exhibited lesions consistent with multifocal moderate to severe bronchio-interstitial pneumonia with vasculitis (Figure 15D–F). The one mouse that died following inoculation with SARS-CoV-2-[Orf3a-ΔYxxФ] exhibited lung lesions similar to mice inoculated with SARS-CoV-2 (Figure 15H–I). Taken together, these results indicate that removing the three potential tyrosine-based sorting motifs in ORF3a resulted in the decreased pathogenicity of SARS-CoV-2 in K18-hACE-2 mice.

## 4. Discussion

Previous studies have shown that the ORF3a protein was a virulence factor in SARS-CoV-2 pathogenesis [39]. Using the hACE-2/mouse model, the deletion of ORF3a from SARS-CoV-2 did not result in a significant reduction in virus titers in cell culture but had a profound effect on lung pathology [39], indicating that the ORF3a contributes to the pathogenesis caused by SARS-CoV-2. ORF3a has several reported biological functions that could impact the pathogenesis in the hACE-2/mouse model. These functions include an ion channel activity, the ability to induce apoptosis, the disruption of the autophagy pathway, causing membrane perturbations, the activation of the NLRP3 inflammasome pathway, and alterations in virus egress [3,40,41,42,43,44,45,46,47,48]. Thus, the identification of those protein domains that are important to ORF3a trafficking to different cellular compartments and their association with different biological functions in vivo could aid in the development of more robust vaccines that result in long-term immunity against this viral disease.

In a recent study, the SARS-CoV was shown to cause apoptosis through both the intrinsic and extrinsic pathways, while the SARS-CoV-2 ORF3a caused apoptosis through an extrinsic pathway [31]. This result was based on the findings that the presence of SARS-CoV-2 ORF3a resulted in the cleavage of pro-caspase 9 and pro-caspase 8 of the extrinsic pathway [31]. These investigators showed that a tyrosine-to-alanine substitution within the tyrosine-based sorting motif (^160^YNSV^163^) resulted in a protein (ORF3a-YA) that no longer trafficked to the cell-plasma membrane and did not induce apoptosis, suggesting that cell-surface expression was necessary for SARS-CoV-2 ORF3a-induced apoptosis. However, these investigators also showed that ORF3a-YA was not associated with cell-membrane fractions. The reason for this finding is unknown, since this tyrosine-to-alanine substitution should not affect the biosynthesis and translocation of this protein across the RER membrane. Finally, these investigators did not analyze the other potential tyrosine-based sorting motifs within the ORF3a cytoplasmic domain.

In this study, we have expanded on the studies of the tyrosine-based motifs of the SARS-CoV-2 ORF3a protein. A sequence analysis of the cytoplasmic domain (CD) of ORF3a proteins from SARS-CoV-2 isolates revealed that more than one potential tyrosine-based sorting motif exists in the CD of these proteins (^160^YNSV^163^, ^211^YYQL^214^, and ^233^YKNI^236^) and these motifs are highly conserved. Thus, the studies presented here are unique, as we addressed whether other potential tyrosine-based sorting motifs contribute to the trafficking of the SARS-CoV-2 ORF3a to the cell surface, and if so, do they also contribute to the biological functions of this protein? We used a strategy in which we first eliminated all three tyrosine-based sorting signals by altering each tyrosine to an alanine. The resulting protein, ORF3a-[ΔYxxΦ], was analyzed for intracellular trafficking by co-transfecting plasmids expressing proteins tagged with fluorescent proteins that localize in different organelles or antibodies specific to organelle-specific proteins. ORF3a-[ΔYxxΦ] was detectable in several intracellular compartments of the secretory pathway organelles (ER, ERGIC, cis-medial Golgi, trans-Golgi, and TGN) but was not detected at the cell surface. In contrast, the unmodified ORF3a was readily detected at the cell surface. Several biological functions attributed to ORF3a including apoptosis, incomplete autophagy, the activation of the NLRP3 inflammasome, and the possible use of lysosomes as a pathway for virus egress are accomplished by trafficking to the cell-plasma membrane, followed by endocytosis to the late endosomes/lysosomes [40,41,42,43,44,45,46,47,48]. Following endocytosis, ORF3a interacts with Vps39 of the homotypic fusion and protein-sorting (HOPS) complex, a multi-protein tethering complex crucial for the fusion of endosomes and autophagosomes to lysosomes [49]. Thus, ORF3a prevents the fusion of lysosomes and late endosomes, which causes incomplete autophagy [42,43,44]. While the fusion of autophagosomes with lysosomes and late endosome fusion with lysosomes use similar but distinct pathways, they use some of the same components, and these pathways likely intersect. In a more recent study, ORF3a leads to the hyperactivation of the late endosomal and lysosomal Rab7, interacts with Vps39, and sequesters the Rab7 GAP TBC1D5. This interaction leads to a displacement of Rab7 from this complex and the disruption of the GTP hydrolysis cycle of Rab7 [50]. How these two pathways may influence ORF3a-mediated fusion of autophagosomes and lysosomes is still unknown. Our confocal microscopy studies/immunofluorescence studies of cells revealed no co-localization of ORF3a-[ΔYxxΦ] with LAMP-1 in Z-stacks of cells. Using a Lyso-IP technique to fractionate late endosomes and lysosomes from cells, we showed using immunoblots and lysosome markers that cathepsin-D, LAMP-1, and LAMP-2 were detected in Lyso-IP fractions, while cytosol, mitochondrial, and Golgi markers were not detected. The unmodified ORF3a was detected in the late endosome lysosome fraction, while ORF3a-[ΔYxxΦ] was not detected in the late endosome/lysosome fractions. The results from the viral growth curves of the different mutants suggest that the SARS-CoV-2 expressing the unmodified ORF3a and the SARS-CoV-2-(ORF3a-[ΔYxxΦ] grew to similar titers based on levels of infectious virus released. Combined with our immunofluorescence results showing that the ORF3a-[ΔYxxΦ] was not detected in lysosomes, it would suggest that virus egress via lysosome exocytosis in cell culture may not be a major pathway for infectious virus release. The Orf3a-[ΔYxxΦ] mutant also has implications for the activation of the NLRP3 inflammasome by the ORF3a protein. In a recent publication, Xu and colleagues showed that ORF3a activated the NLRP3 inflammasome [6]. In their model, they propose that the ORF3a is transported to the cell surface and, through its ion channel activity, causes the efflux of K+ ions from the cell, which is known to activate the NLRP3 inflammasome [6]. As ORF3a-[ΔYxxΦ] is not transported to the cell-plasma membrane, it will be of interest to determine if the ORF3a-[ΔYxxΦ] can still activate the NLRP3 inflammasome.

Unlike previous studies, we analyzed all three potential tyrosine-based sorting motifs for transport to the cell surface. We constructed a series of mutants with one or two tyrosine-based sorting motifs intact to determine the importance of each tyrosine motif alone or the combinations of two motifs. If the ^160^YNSV^163^ motif alone was responsible for the transport to the surface, the mutant with just the ^160^YNSV^163^ motif intact (ORF3a-[Y160]) should be transported to the cell surface. Our results indicated that ORF3a-[Y160] was not transported to the cell surface, indicating that the ^160^YNSV^163^ motif likely does not dictate efficient transport to the cell surface. Our results also show that the ORF3a with the ^211^YYQL^214^ motif (ORF3a-[Y211]) was transported to the cell surface. Finally, our data also indicate that mutant ORF3a-[Y160,211] was transported to the cell surface at much lower levels, indicating that the presence of the ^160^YNSV^163^ motif did not significantly interfere with transporting ORF3a to the cell surface or that the ^160^YNSV^16^ motif assisted in transporting ORF3a to the cell surface. It should be noted that another mutant, ORF3a-[Y211, 233], the equivalent to the Y160A mutant analyzed by Ren and colleagues [31], was also not detected at the cell surface. The inability of the ORF3a-[Y211, 233] to be transported to the cell surface may have to do with the adaptor protein complexes that bind to the tyrosine-based sorting signals. Transmembrane protein trafficking is a highly dynamic and efficient process regulated by a network of proteins with AP complexes (AP-1 to AP-5) as key regulators of this process. Each AP complex performs the specific sorting function at distinct intracellular organelles and the sorting signals recognized by AP complexes are located in the cytoplasmic domain. These signals consist of short, linear sequences of amino-acid residues. The best-characterized sorting signals are tyrosine-based (YXXØ) (X is any amino acid and Ø is a bulky hydrophobic amino acid, i.e., leucine, isoleucine, methionine, valine, or phenylalanine), which binds with μ1–μ3 subunits of AP-1 to AP-3 and the dileucine-based ([DE]XXXL[LI]) signals, which are not present in the ORF3a protein. The reviewer is correct regarding the localization of the HA-ORF3a-[Y211,233] mutant, and initially we thought this mutant with its ^211^YYQL^214^ would have been trafficked to the cell surface. However, this was not the case. One potential explanation for this observation is that changing the tyrosine to alanine in the ^160^YNSV^163^ motif resulted in ORF3a binding with another AP complex and trafficking to another intracellular organelle. Alternatively, the different tyrosine-based sorting motifs may bind to different AP complexes. The removal of one motif may allow for a stronger binding to another AP complex. While this study was confined to the analysis of ORF3a sequences from SARS-CoV-2, it should be pointed out that SARS-CoV also has three tyrosine-based sorting signals (^160^YNSV^163^, ^200^YVVV^203^, and ^211^YYQL^214^) [Supplementary Figure S1].

To emphasize the importance of the tyrosine-based motifs in Orf3a function and pathogenicity, we performed pathogenicity studies using K18hACE2 mice. The inoculated K18hACE2 mice were inoculated with either the SARS-CoV-2 expressing the unmodified ORF3a gene or with SARS-CoV-2-[ORF3a-[YxxΦ] and were observed for 21 days for weight loss and clinical signs of disease. The mice inoculated with SARS-CoV-2 developed clinical signs of disease and loss of body weight, resulting in the euthanasia of five of eight mice at 7–8 days post-infection. These mice exhibited histopathology similar to other published studies using this strain of mice [39,50,51,52,53]. The lung pathology observed in the mice was graded on a scale of 1 to 4, with a 1 being <10% and a 4 being >50%. Unlike the unmodified virus, the virus expressing the ORF3a-[ΔYxxΦ] was less pathogenic in mice, with only one mouse euthanized at day 8 post-infection. This mouse was euthanized at day 8 with a loss of body weight and clinical signs of disease. The pathology score of this ORF3a-[ΔYxxΦ]-inoculated mouse was 2. Thus, examining the pathogenicity of viruses expressing the other modified ORF3a proteins from this study should provide novel information on the role of these potential tyrosine-based sorting motifs in the pathogenicity of SARS-CoV-2. Information on an attenuated ORF3a protein could be used with other SARS-CoV-2 proteins attenuated for virulence in a next-generation live-attenuated, non-pathogenic vaccine that would generate immunity to additional virus proteins.

## 5. Conclusions

In this study, we examined the role of the three potential tyrosine-based sorting motifs (^160^YNSV^163^, ^211^YYQL^213^, and ^233^YNKI^236^) in the intracellular trafficking of the open reading frame 3a protein from SARS-CoV-2. All three motifs are highly conserved in SARS-CoV-2 strains but only the ^160^YNSV^163^ motif has been analyzed for the intracellular transport of the ORF3a protein to the cell surface. We have generated a series of ORF3a mutations in which tyrosine from one, two, or all three motifs were substituted with alanine residues. Our results indicate that the ^211^YYQL^213^ motif was most important for efficient transport of ORF3a to the cell-plasma membrane. Additionally, we showed that the removal of all three tyrosine-based sorting signals (ORF3a-[ΔYxxΦ] was less stable, did not co-localize with the LAMP-1 expressing late endosomes/lysosomes, and a SARS-CoV-2 virus expressing ORF3a-[ΔYxxΦ] was less pathogenic than the unmodified SARS-CoV-2 virus in mice expressing the human angiotensin-converting enzyme II (ACE-II), a receptor for SARS-CoV and SARS-CoV-2.

## Figures and Tables

**Table 1:** Primers used for amplification of SARS-CoV-2 subgenomic fragments and the BAC-YAC vector.

**Fragment**	**Primer**	**Sequence (5′–3′)**	**Size (bp)**	**Overlap with Next Fragment (bp)**
1	Delta-F1-F	ATATTAGGTTTATACCTTCCCAGG	3317	276
	Delta-F1-R	CTGGTGTAAGTTCCATCTCTAATTG		
2	Delta-F2-F	CAGATGAGGATGAAGAAGAAGG	3271	313
	Delta-F2-R	TTTGTGCTCCAAAGACAACGTATAC		
3	Delta-F3-F	ATCTTGTACCAAACCAACCATATCC	3016	301
	Delta-F3-R	TCAGCAGCCAAAACACAAGC		
4	Delta-F4-F	GTGACATAGCATCTACAGATACTTG	3249	303
	Delta-F4-R	CTAAGAGAATGTCATTGTGTAACTGG		
5	Delta-F5-F	TCAACCGCTACTTTAGACTGAC	2940	266
	Delta-F5-R	AATAGATTACCAGAAGCAGCGTG		
6	Delta-F6-F	AACTGTTTGGATGACAGATGC	3267	206
	Delta-F6-R	AACCAAAGCACTCACAGTG		
7	Delta-F7-F	ATGCCAGATTACGTGCTAAGCAC	2960	249
	Delta-F7-R	ACCTAACTGACTATGACTAAAATCTC		
8	Delta-F8-F	GGAGTCACATTAATTGGAGAAGC	3171	291
	Delta-F8-R	GCATCAGTAGTGTCAGCAATGTC		
9	Delta-F9-F	ACCTTGTAATGGTGTTGAAGG	2945	346
	Delta-F9-R	TCATGTTCAGAAATAGGACTTGTTG		
10	Delta-F10-F	GATGGCAACTAGCACTCTCC	3178	204
	Delta-F10-R	TTTGGCAATGTTGTTCCTTGAGG		
11	Delta-F11-F	GAAAGATCTCAGTCCAAGATGG	1301	123
	Delta-F11-R	TTTTTTGTCATTCTCCTAAGAAGCT		
BAC-YAC	BAC-YAC-F	CGAGTGTACAGTGAACAATGC	9016	64
	BAC-YAC-R	GAACAGATCTACAAGAGATCG		

**Table 2:** Primers used for site-directed mutagenesis of ORF3a.

Primer	Sequence (5’–3’)
Y160A-F	CTTTGCTGGCATACTAATTGTTACGACTATTGTATACCTGCAAATAGTGTAACTTCTTCATAGGGATAACAGGGTAATCG
Y160A-R	ACCTGAAGTAATGACAATTGAAGAAGTTACACTATTTGCAGGTATACAATAGTCGTAAGCCAGTGTTACAACCAATTAAC
Y211A-F	AAAGACTGTGTTGTATTACACAGTTACTTCACTTCAGACGCATACCAGCTGTACTCAACTTAGGGATAACAGGGTAATCG
Y211A-R	AGTGTCTGTACTCAATTGAGTTGAGTACAGCTGGTATGCGTCTGAAGTGAAGTAACTGGCCAGTGTTACAACCAATTAAC
Y233A-F	AGTACAGACACTGGTGTTGAACATGTTACCTTCTTCATCGCAAATAAAATTGTTGATGAGTAGGGATAACAGGGTAATCG
Y233A-R	TTGGACATGTTCTTCAGGCTCATCAACAATTTTATTTGCGATGAAGAAGGTAACATGTGCCAGTGTTACAACCAATTAAC

## Data Availability

Data is contained within the article or Supplementary Materials.

## Supplementary Materials

The following supporting information can be downloaded at: https://www.mdpi.com/article/10.3390/v17040522/s1, Figure S1. Seventeen ORF3a sequences from SARS-CoV (strain Tor2) and SARS-CoV-like strains. Figure S2. Body weights of female and male mice inoculated with the vehicle control, SARS-CoV-2 [Orf3a-ΔYxxP], or the unmodified virus SARS-CoV-2.
