# Supplementary material for: The Role of the Tyrosine-Based Sorting Signals of the ORF3a Protein of SARS-CoV-2 in Intracellular Trafficking and Pathogenesis
Source: Viruses. 2025 Apr 3;17(4):522. doi: 10.3390/v17040522 (PMC12031507; doi:10.3390/v17040522)
Supplement: Supplementary file 1 [file viruses-17-00522-s001.zip › viruses-3546465-supplementary.pdf]

```
                   160                                      200             211                              233
Tor2               DYCIPYNSVTDTIVVTEGDGISTPKL---KDYVVVHGYFTEVYYQLESTQITTDTGIENATFFIFNKLVKDP 240
Civet007           DYCIPYNSVTDTIVVTEGDGISTPKL---KDYVVVHGYFTEVYYQLESTQITTDTGIENATFFIFNKLVKDP 240
RsYN03             DYCIPYNSVTDTIVVTSSDGTNVPKL---KDYVVIHGYFTEIYYQLESTQLSTDTGAENATFFIYSKLVKDA 240
Rp/Shaanxi2011Ba   DYCIPYNSITDTIVLTSGDGTNQPKL---KDYVVIHGYFTEVYYQLETTQLSPETGVENATFFIFSKLVKAV 240
Rf1/2004           DYCIPYNSVTDTIVVTSGDGISTPEL---KDYVVVHGYFTEVHYQLESTQITTDTGIQNATFFIFNKLVKDP 240
Rf-HeB2013         DYCIPYNSVTDTIVVTSGDGISTPEL---KDYVVVHGYFTEVHYQLESTQITTDTGIQNATFFIFNKLVKGP 240
BtRf-JL2012        DYCIPYNSVTDTIVVTTGDGISTPEL---KDYVVVHGYFAEVHYQLESTQITTDTGIQNATFFIFNKLVKDP 240
HKU3-1             DYCIPYNSVTDTVVITSGDGTNQPKL---KDYVVIYGYFTEVYYQLESTQLSTDTGAENATFFIYSKLVKDV 240
LYRa11             DYCIPYNSVTDTIVLTSSDGTNVPKL---KDYVVIHGYFTEIYYQLESTQLSTDTGAENATFFIYSKLVKDA 240
Rs9401             DYCIPYNSVTDTIVVTAGDGISTPKL---KDYVVVHGYFTEVYYQLESTQITTDTGIENATFFIFNKLVKDP 240
Rp3/2004           DYCIPYNSITDTIVLTSGDGTTQPKL---KDYVVIHGYFTEVYYQLESTQLSTDTGAENATFFIYSKLVKDV 240
WIV16              DYCIPYNSVTDTIVVTAGDGISTPKL---KDYVVVHGYFTEVYYQLESTQITTDTGIENATFFIFNKLVKDP 240
RsSHC014           DYCIPYNSVTDTIVVTAGDGISTPKL---KDYVVVHGYFTEVYYQLESTQITTDTGIENATFFIFNKLVKDP 240
WIV1               DYCIPYNSVTDTIVVTAGDGISTPKL---KDYVVVHGYFTEVYYQLESTQITTDTGIENATFFIFNKLVKDP 240
RsYN09             DYCIPYNSVTDTIVLTSSDGTNVPKL---KDYVVIHGYFTEIYYQLESTQLSTDTGAENATFFIYSKLVKDE 240
YNLF_34C           DYCIPYNSVTNTIVVTAGDGISTPEL---KDYVVVHGYFTEVHYQLESTQISTDTGIQNATFFIFNKLVKDP 240
Rs3367             DYCIPYNSVTDTIVVTAGDGISTPKL---KDYVVVHGYFTEVYYQLESTQITTDTGIENATFFIFNKLVKDP 240
```

Supplementary Figure S1. Seventeen ORF3a sequences from SARS-CoV (strain Tor2) and SARS-CoV-like strains (all 274 amino acids in length) from amino acid 155 to 240 showing the potential tyrosine-based sorting motifs (in red).

Supplementary Figure S2. Body weights of female mice (F1-4) or male mice (M1-4) inoculated with the DMEM vehicle control (Panel A), SARS-CoV-2 [Orf3a-ΔYxxP] (Panel B), or the unmodified virus SARS-CoV-2 (Panel C). The experiment was run for 21 days with 8 mice per group. Body weights are presented as a percentage of the body weight at day 0. At 21 days the experiment was terminated and mice euthanized. Mice that were euthanized in poor health were Panel B, (F2); Panel C (F4, M1-3)

Day post-infection

A.

	0	1	2	3	4	5	6	7	8	9	10	11	12	14	16	18	21
F1	100	98.2	100.3	100.6	103.5	103.3	106.1	106.7	106.2	107.6	104.3	104.1	106.6	107.7	105.1	107.2	103.8
F2	100	101.2	100.8	101.4	103.1	103.7	105	105.2	106.4	107.6	106	108.1	110.9	108	105.1	113.2	111
F3	100	99.7	99.4	101	101	101.7	101.9	101.3	100.2	102.4	102.4	100.2	103.2	100.4	104.6	104.2	104.9
F4	100	101.2	101	101.4	101.8	96.9	94.9	99.1	100.6	103.7	104.4	104	105.1	103.3	104.4	104.6	104.2
M1	100	100.5	100.8	101.8	102.6	102.6	104.9	106.8	111.4	112	113.7	115.7	116.2	117.1	120.8	123.1	120.7
M2	100	101.8	102.1	103.6	105.2	106.4	107.2	107.5	108.5	108.9	110.7	111.8	112.9	113.6	114.7	118.5	113.9
M3	100	99.3	103	103.7	99.4	104.2	104	106.4	107.6	109.1	108.4	108.9	111	109.9	112.6	114.7	109.3
M4	100	100.1	100.9	101.2	101.8	103.3	102.7	103.9	105.2	106.3	105.2	106.3	106.9	109.4	110.1	112.2	110.6

B.

	0	1	2	3	4	5	6	7	8	9	10	11	12	14	16	18	21
F1	100	99.7	99.9	98.9	101.3	101.1	104.1	105	103	106.8	106.2	107.7	107.4	107.5	105.5	107.4	108.7
F2	100	99.9	101.3	100.7	101	99.9	95.5	88.9	82.4 †	-	-	-	-	-	-	-	-
F3	100	98.6	99.4	98.5	98.6	98.6	101.5	101	101.2	102.1	100.7	100.1	103.4	103.4	106.5	103.5	108
F4	100	97.7	97.1	96.4	96.9	97.6	98.7	98.6	102.3	102.2	101.1	99.6	99.9	99.9	101	103.8	104.8
M1	100	97.2	98.5	98.7	98.4	101.7	102.9	101.3	100.4	103.2	102.7	103.8	103.8	103.4	104.8	108.8	107.9
M2	100	99.2	99.3	100.3	102.5	106.4	103.2	103.7	104.6	103.5	102.5	102.2	104.4	103.4	103	104.9	103.4
M3	100	99.7	99	99.7	100.5	100.1	102.6	103.3	102.7	105	103.3	105	104.3	108.5	105.5	106.7	106.5
M4	100	99.5	101.5	101	102.7	104.6	106.6	107.8	109.2	109.3	109.1	109.2	112.4	116	116.8	120	123.5

C.

	0	1	2	3	4	5	6	7	8	9	10	11	12	14	16	18	21
F1	100	97.6	98.6	99.1	99.3	96.8	87.2	82.5	76.5 †	-	-	-	-	-	-	-	-
F2	100	96.6	100.3	101.6	103	100.1	88.7	105.2	90.1	98.5	103	105.1	105.9	104.4	103.4	104.7	105.1
F3	100	98.3	102	101.7	101.7	93.6	86.2	80.5	86.1	93.5	98.8	100.8	104.2	102.4	97.3	98.8	99.4
F4	100	101.7	103.5	102.9	101.3	95.3	86.4	80.3 †	-	-	-	-	-	-	-	-	-
M1	100	97.8	99	100.6	100.8	92.8	86	79.8 †	-	-	-	-	-	-	-	-	-
M2	100	99.4	101.1	101	101	92	84.4	79 †	-	-	-	-	-	-	-	-	-
M3	100	98.4	97	100	100.2	104	94.4	88.1 †	-	-	-	-	-	-	-	-	-
M4	100	98.2	99.3	100.6	101.2	100	88.7	94.6	100.4	103.4	103.9	104.9	106.8	108	110.2	112.6	115.4
